# Inversion Polymorphisms Carried by Introduced Rainbow Trout Disrupt Hybrid Zones Between Native Species

**DOI:** 10.1111/mec.70556

**Published:** 2026-09-20

**Authors:** Craig D. Wells, Seth R. Smith, Marty Kardos, Stephen J. Amish, Angela Lodmell, Sally Painter, Brian Stephens, James Dunnigan, Scott Relyea, Jason Nachtmann, Paul A. Hohenlohe, Matthew C. Boyer, Ryan P. Kovach, Andrew R. Whiteley

**Affiliations:** ^1^ Bioinformatics and Computational Biology University of Idaho Moscow Idaho USA; ^2^ Wildlife Biology Program, W.A. Franke College of Forestry and Conservation University of Montana Missoula Montana USA; ^3^ Flathead Lake Biological Station, Division of Biological Sciences University of Montana Polson Montana USA; ^4^ Northwest Fisheries Science Center, National Marine Fisheries Service National Oceanic and Atmospheric Administration Seattle Washington USA; ^5^ Department of Biology, Graduate Degree Program in Ecology Colorado State University Fort Collins Colorado USA; ^6^ The University of Montana Fish and Wildlife Genomics Laboratory Missoula Montana USA; ^7^ Montana Fish, Wildlife & Parks Libby Montana USA; ^8^ Sekokini Springs Hatchery Montana Fish, Wildlife & Parks Kalispell Montana USA; ^9^ Murray Springs Hatchery Montana Fish, Wildlife & Parks Eureka Montana USA; ^10^ Department of Biological Sciences University of Idaho Moscow Idaho USA; ^11^ Montana Fish, Wildlife & Parks Kalispell Montana USA; ^12^ Montana Fish, Wildlife & Parks Missoula Montana USA

**Keywords:** hybrid swarm, incompatibility, *Oncorhynchus lewisi*, *Oncorhynchus mykiss gairdneri*, recombination suppression, reproductive isolation, selection, structural variation

## Abstract

Genomic mechanisms explaining why some hybrid zones remain stable while others collapse into hybrid swarms are poorly understood. In Montana's Kootenai River Basin, native interior redband trout ( *Oncorhynchus mykiss gairdneri* ; IRT) and westslope cutthroat trout ( *O. lewisi* ; WCT) are naturally sympatric and historically exhibited limited hybridization. However, the introduction of allopatric coastal rainbow trout (*O. m.* ssp.; CRT) has coincided with hybrid swarm formation. Using novel IRT linkage maps and 3489 individuals genotyped at 189,984 SNPs, we characterized the role of genome structure in hybridization among native and invasive taxa. Native IRT were fixed for the derived arrangement of a double inversion complex on chromosome 5 (Chr05), whereas introduced CRT and intraspecific hybrids retained the ancestral polymorphism associated with migratory behaviour. Widespread hybridization was primarily driven by intraspecific admixture among rainbow trout (RBT) taxa, rather than with other species, likely mediated by genomic structural variants introduced into IRT populations via CRT admixture. Specifically, Chr05 genotypes exhibited strong habitat stratification; migratory‐associated haplotypes occurred primarily in migration‐associated habitats, implicating dispersal as a key mechanism promoting introgression. We found evidence of selection against interspecific hybrids, possibly tied to interspecific inversions. Notably, WCT carried a distinct form of Chr05 that is colinear with the ancestral RBT form. Interspecific hybrids carrying both the WCT and ancestral RBT forms (WCT_A_/RBT_A_), despite being structurally homozygous, were rare (1.59%), suggesting selection or stronger genomic incompatibility. Ultimately, introduced intraspecific variation may facilitate introgression via dispersal while distinct interspecific structural arrangements maintain genomic barriers, restricting gene flow.

## 1 Introduction

Hybridization between native and non‐native taxa can reduce fitness via outbreeding depression and potentially lead to the extinction of distinct native lineages. Hybrid zones between naturally sympatric taxa are often stable and maintained by a combination of pre‐ and post‐zygotic barriers that reduce the frequency of hybridization and hybrid fitness, allowing parental lineages to remain distinct with limited introgression (Harrison 1993; Allendorf et al. 2001; Coyne and Orr 2004). In contrast, hybridization following secondary contact between historically allopatric taxa can facilitate introgression when reproductive barriers are incomplete (Allendorf et al. 2001). Human‐mediated translocations are of particular conservation concern because they often introduce allopatric lineages that lack co‐evolutionary history with native taxa, potentially increasing the likelihood of hybrid swarm formation and genomic extinction relative to instances of naturally occurring secondary contact (Leary et al. 1985; Abbott et al. 2016; Todesco et al. 2016; Kovach et al. 2016, 2025). Salmonid fishes provide a well‐studied system for examining these dynamics. In Pacific trout (*Oncorhynchus*), hybridization between and within rainbow trout ( *O. mykiss* ; RBT) and cutthroat trout ( *O. clarkii* sensu lato) has been especially consequential, obscuring evolutionary history (Thorgaard et al. 2018) and complicating conservation and management (Kovach et al. 2025).

RBT are a phylogenetically complex taxon comprising deeply diverged lineages (Campbell et al. 2023; Hale et al. 2024). Historically, coastal rainbow trout (*O. m. irideus*) and related subspecies (e.g., *O. m. aquilarum*) were confined to Pacific coastal and Great Basin drainages (Busby et al. 1996; Behnke 2002; Simmons 2011). Populations from coastal lineages were used extensively to produce common California‐origin (USA) hatchery strains (Leary et al. 2008), herein referred to as CRT (*O. m*. ssp.). A distinct RBT subspecies, interior redband trout (*O. m. gairdneri*; IRT), is native to portions of the Columbia River drainage east of the Cascade Mountains and to the upper Fraser and Athabasca River drainages of western North America (Behnke 2002; Taylor et al. 2007). Native IRT naturally co‐occur with westslope cutthroat trout ( *O. lewisi* ; WCT) throughout much of the upper Columbia River drainage, where these taxa have long existed in sympatry. Despite this overlap, IRT and WCT appear to have evolved natural isolation mechanisms (Allendorf et al. 2005; Campbell et al. 2013), potentially mediated by prezygotic mechanisms such as differences in life history and spawning behaviour (Rubidge and Taylor 2004). Postzygotic mechanisms that could contribute to isolation in these taxa remain poorly understood. The introduction of CRT—which are naturally allopatric with both IRT and WCT (Leary et al. 2008)—has further complicated hybridization dynamics as CRT readily hybridize with native trout following introduction (Weigel et al. 2003). Natural sympatry between WCT and native IRT does not imply ecological segregation and instead reflects the maintenance of reproductive barriers that are notably absent in introduced CRT (Kovach et al. 2017). Consequently, CRT act as a distinct genetic threat to native taxa, potentially eroding the reproductive isolation observed among native trout species (Weigel et al. 2003; Kovach et al. 2017).

Across river systems with RBT × WCT hybridization, studies have consistently shown that environmental and landscape variables are correlated with the extent and direction of introgression. Warmer temperatures, altered hydrology, increased connectivity, and stocking history appear to be associated with increased hybridization and hybrid swarm formation (Muhlfeld, McMahon, Boyer, and Gresswell 2009; Rasmussen et al. 2010; Yau and Taylor 2013; Muhlfeld et al. 2014, 2017; Kovach et al. 2016; Young et al. 2016). While much of this work has emphasized ecological correlates of hybridization, comparatively little attention has been paid to the potential role of genome structure or intraspecific variation within RBT. Studies attempting to distinguish IRT from CRT often relied on a small number of allozyme loci (Allendorf and Utter 1979; Allendorf et al. 1980; Utter and Stefan 1984; Williams et al. 1996; Utter 2000; Knudsen et al. 2002) or reduced single nucleotide polymorphism (SNP) panels (Zambie et al. 2026). Therefore, the extent of intraspecific RBT hybridization, and how large‐scale structural genomic variation may shape patterns of introgression, is not understood.

Advances in genomic resources have greatly improved the resolution with which hybrids can be detected, and structural variation is increasingly recognized as an important factor shaping hybridization outcomes in conservation‐relevant systems (Galbusera et al. 2025; Pegan et al. 2025; Schneller et al. 2025). Genome assemblies for various RBT lineages (Gao et al. 2021; Ali et al. 2025, 2026), along with assemblies and a linkage map for WCT, have revealed numerous Robertsonian rearrangements and large inversions that differ within and between taxa (Flores et al. 2025). Structural variation can strongly influence recombination, introgression, and hybrid fitness (Ostberg et al. 2013; Wang et al. 2025). While inversions between RBT and WCT are putatively fixed (Flores et al. 2025), polymorphic inversions exist within RBT (Pearse et al. 2019; Hale et al. 2024). Of particular interest is a roughly 57 Mb double inversion on chromosome 5 (Omy05; herein the Chr05 complex), which is polymorphic within RBT and contributes to explaining variation in life‐history, growth, and development (Pearse et al. 2019; Rundio et al. 2021; Kobayashi et al. 2024). The ancestral haplotype of this inversion (RBT_A_) is colinear with WCT (Flores et al. 2025), potentially facilitating higher recombination in RBT × WCT hybrids. The distribution and evolutionary significance of such polymorphisms in IRT remain incompletely characterized, but fixed inversions between RBT and WCT and polymorphic inversions within RBT may help explain why hybrid swarms form in some contact zones but not others.

Here, we present novel linkage maps for IRT native to the Kootenai (spelled ‘Kootenay’ in Canada) River Basin and combine genome‐wide SNP data with existing lineage‐specific genome assemblies to resolve ancestry and structural genomic variation in a multispecies hybrid complex across the Kootenai River Basin in Montana (USA). We identify taxon‐specific diagnostic loci distinguishing native IRT and WCT alongside introduced CRT and Yellowstone cutthroat trout (*O. virginalis bouvieri*; YCT). Additionally, we characterize fixed interspecific rearrangements and polymorphic inversions within RBT, genotype these structural variants across hybrid individuals, and assess signatures of recombination suppression and hybrid incompatibility. Using this framework, we (1) describe genome structure of Kootenai River IRT, (2) characterize landscape‐scale patterns of hybridization among IRT, CRT, WCT, and YCT, (3) evaluate how structural variants shape hybrid ancestry and recombination, and (4) assess how genome structure may contribute to asymmetric hybridization outcomes. We also reveal potential patterns of selection and incompatibility between RBT and WCT mediated by complex structural variants, providing possible answers to long‐standing questions in these taxa.

## 2 Methods

### 2.1 Study System

The Kootenai River Basin spans southeastern British Columbia (Canada), northwestern Montana, and northern Idaho (USA), and is the second largest tributary of the Columbia River (Knudson 1994). Its hydrology and connectivity have been heavily modified by Libby Dam (completed 1972), which created Koocanusa Reservoir and blocks upstream fish passage. Historically, the mainstem Kootenai River was dominated by WCT, but following dam construction, RBT became the dominant taxon in the mainstem river (USFWS 1999).

**FIGURE 1:**
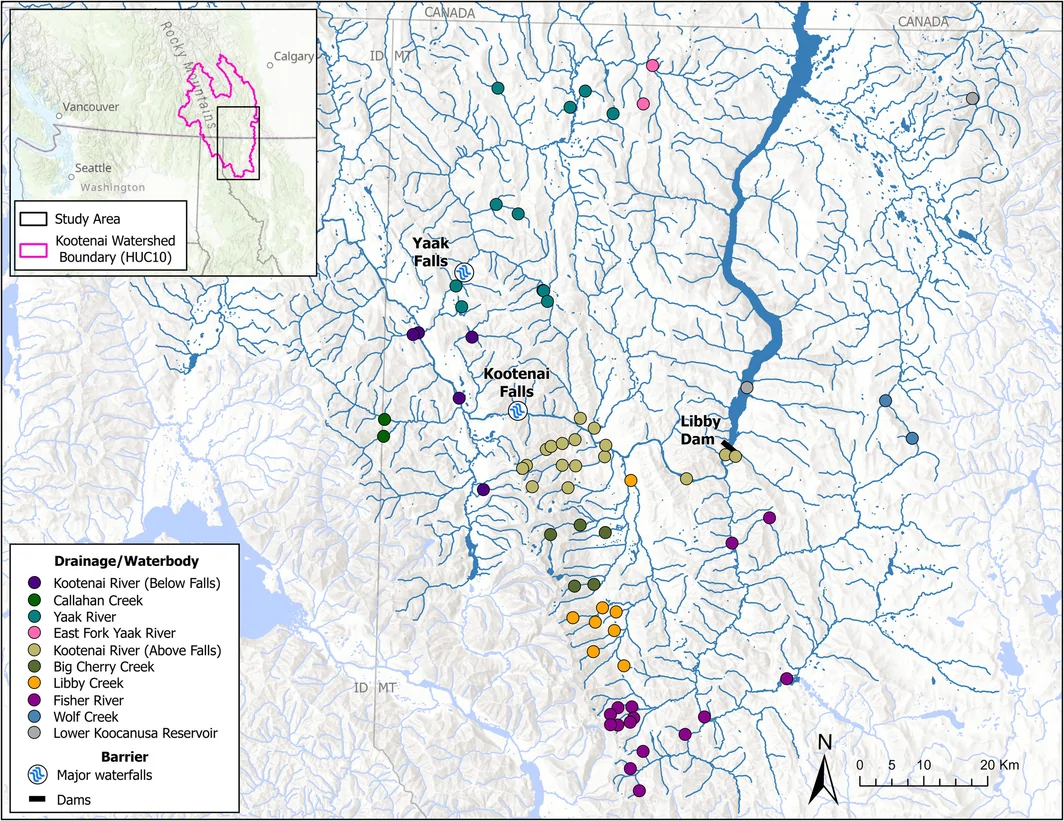
Map of sampling locations for *Oncorhynchus* collections across the Kootenai River Basin, Montana, USA. Sites are colour‐coded by subbasin drainage. Major hydrologic barriers separating key drainages (e.g., Libby Dam, Kootenai Falls, Yaak Falls) are indicated. Temporally replicated collections are represented by a single point. Sample locations within British Columbia, Canada are not pictured.

Our study focuses on the Montana portion of the basin, including the Yaak River, Libby Creek, and Fisher River subbasins, and Koocanusa Reservoir (Figure 1). IRT historically occurred in fewer waters than WCT; Hensler et al. (1996) concluded that they were confined to parts of the system downstream from the Fisher River drainage. Within this area, IRT occurred in some headwaters as well as downstream reaches, while WCT were more broadly distributed. The native distributions of the two taxa were spatially complex, with IRT and WCT occurring allopatrically in some waters and sympatrically in others. Non‐native RBT (believed to be primarily CRT) and YCT were stocked extensively in Kootenai lakes, tributaries, and reservoirs beginning in the late 1800s (Huston 1999, 2000). YCT stocking ended in the 1960s when hatcheries shifted to using WCT. Stocking of streams ceased in 1974 (Zackheim 2005). Previous allozyme‐based research in the basin suggested these introductions produced asymmetric hybridization outcomes: hybrid zones involving non‐native CRT were more likely to develop into hybrid swarms, whereas hybridization between naturally sympatric taxa (native IRT and WCT) rarely progressed beyond early‐generation hybrids (Leary et al. 2008). Thus, the Kootenai Basin provides an ideal, heavily augmented multispecies system to investigate how genomic architecture may drive these differing introgression outcomes.

### 2.2 Genetic Map Construction and Genome Structure Analysis

At the onset of this study, linkage maps or chromosome‐scale genome assemblies were available for WCT and CRT, but not for IRT. A consensus map and three population‐specific linkage maps for IRT were constructed using LepMap3 (Rastas 2017) using methods comparable to those in Flores et al. (2025) (see Supporting Information).

Per‐population genetic maps and the global consensus map were aligned to the Arlee RBT reference genome (NCBI: GCF_013265735.2; Gao et al. 2021), the Swanson RBT reference genome (NCBI: GCA_025558465.1; Ali et al. 2025), and the Connor Lake WCT reference genome (NCBI: GCF_045791955.1; Flores et al. 2025) using Minimap2 v2.30 (Li 2018, 2021) to identify karyotypic rearrangements and inversions. The Arlee strain is primarily representative of CRT ancestry (northern California origin), whereas the Swanson strain originated from the Kenai Peninsula, Alaska, USA, and represents a northern RBT lineage rather than the California‐origin CRT or IRT lineages considered here. Inversions ≥ 2 Mb in length were targeted to ensure sufficient marker density in downstream analyses. Alignments of the Arlee RBT reference genome to the Connor Lake WCT and the Swanson RBT genomes were performed using the D‐GENIES web server (https://dgenies.toulouse.inra.fr/; Cabanettes and Klopp 2018) to identify inversion coordinates within the Arlee RBT genome for subsequent analyses. The polarity, defined as the ancestral or derived/rearranged orientation (Gozashti et al. 2025; Pegan et al. 2025), of most inversions was inferred from existing literature (e.g., Sutherland et al. 2016; Pearse et al. 2019; Hale et al. 2024) for RBT. The WCT orientation was inferred from known RBT orientations and from alignment to the Atlantic Salmon ( *Salmo salar* ) reference genome (NCBI: GCF_905237065.1) as an outgroup. Where polarities were not already established, we aligned all reference genomes to the Atlantic Salmon genome as an outgroup.

### 2.3 Sample Collection

We analysed 3641 unique *Oncorhynchus* samples from throughout the Kootenai River Basin, together with reference and comparison populations from elsewhere in western North America, to capture IRT genetic diversity and represent introduced lineages that act as invasive taxa in the Kootenai River Basin. Fin tissue samples were obtained from tributaries, headwater lakes, the Kootenai River mainstem, and Koocanusa Reservoir in Montana and British Columbia, Canada between 2003 and 2024, primarily through non‐lethal electrofishing conducted by Montana Fish, Wildlife & Parks and the British Columbia Ministry of Water, Land and Resource Stewardship. Fin tissue samples of IRT from the Athabasca River drainage in Alberta, Canada were provided by biologists from Alberta Environment and Protected Areas. The dataset included individuals representing putatively non‐hybridized native IRT and WCT, as well as hybrids involving non‐native CRT and YCT.

To represent potential RBT source lineages, we included multiple hatchery strains of IRT and CRT. IRT hatchery lineages were represented by two strains: Pennask Lake rainbow trout originating from the interior Fraser River drainage in British Columbia and Gerrard rainbow trout which originated from Kootenay Lake, British Columbia. Both were initially considered non‐hybridized representatives of IRT ancestry. CRT hatchery lineages included Arlee strain RBT and Eagle Lake strain RBT sourced from multiple hatcheries. In addition, our dataset included strains of Alberta hatchery RBT. YCT were represented by a collection of range‐wide samples.

### 2.4 Sequencing and Genotyping

All samples were genotyped using a RAD‐Capture panel with 10,000 baits targeting restriction site‐associated DNA (RAD) loci—derived from PstI restriction site RAD sequencing data filtered for putative paralogs—following previously published methods (Ali et al. 2016; Feuerstein et al. 2024; Bell et al. 2026). Sequencing utilized an Illumina HiSeq X with 2 × 150 bp paired‐end reads, targeting 20× minimum coverage. PCR duplicates were removed using *clone_filter* in STACKS v2.68 (Catchen et al. 2013). Reads were then quality trimmed using Trimmomatic v0.39 (Bolger et al. 2014) with default parameters, reverse‐complemented, and demultiplexed using the *process_radtags* module in STACKS. Reads were aligned to the Arlee genome using the BWA‐MEM algorithm in BWA v0.7.17 (Li 2013) with default parameters. All alignments were processed with SAMtools v1.15 (Danecek et al. 2021). BAM files were sorted by genomic coordinate and filtered to remove unmapped reads, secondary alignments, and reads with mapping quality < 20. Filtered BAMs were split and processed by individual chromosome (*N* = 32) to improve genotyping speed, reduce memory usage, and allow parallel processing (GNU Parallel; Tange 2024).

Genotypes were called in STACKS using the *gstacks* module with default parameters aside from *–model marukihigh*, *–var‐alpha* 0.001, and *–gt‐alpha* 0.001. Per‐chromosome VCF files were generated using the *populations* module in STACKS with default parameters, a single population popmap, and no explicit data filtering. All VCF files were sorted and indexed using BCFtools v1.19 (Li 2011) and then concatenated into a single VCF. The merged VCF was normalized against the Arlee reference genome using BCFtools. Genotype‐level filters were applied using VCFtools v0.1.16 (Danecek et al. 2011), with genotypes of quality < 30 or depth < 10 recoded as missing. Locus‐level filtering was performed to retain biallelic SNPs with a minor allele count ≥ 3 and a maximum of 20% missing data per site. We did not filter SNP loci based on linkage disequilibrium (LD) or Hardy–Weinberg equilibrium. Departures from Hardy–Weinberg expectations can result from the strong population structure and admixture present in this system, while admixture and chromosomal rearrangements can generate biologically meaningful LD (Hemstrom et al. 2024). Traditional LD pruning can also preferentially remove loci with large allele‐frequency differences among populations rather than only physically linked loci, potentially biasing analyses of population structure and differentiation (Li et al. 2019; Bercovich et al. 2025). Similar concerns have led investigators to retain unpruned SNP panels in interspecific ancestry analyses (Merondun et al. 2024). Furthermore, reducing LD would remove correlated marker blocks associated with chromosomal rearrangements, which are a primary focus of this study. Accordingly, the unpruned dataset was retained for all downstream analyses. Sensitivity analyses evaluating the effects of LD pruning are reported in the Supplementary Methods and Results. Per‐locus depth metrics were used to identify and remove samples with > 30% missing genotypes. Samples included to ensure quality control were removed (*N* = 152), resulting in the final dataset.

### 2.5 Identification of Reference Groups for Diagnostic Locus Discovery

Reference groups were identified following methods adapted from Mattucci et al. (2019). Genetic structure was explored using genotype‐based PCA in R v4.2.3 (R Core Team 2019) with adegenet v2.1.11 (Jombart 2008; Jombart and Ahmed 2011), and assignments were refined using 10 replicates of unsupervised ADMIXTURE v1.3.0 (Alexander et al. 2009) at *K* = 2. Individuals with a mean global ancestry proportion (*Q*) of ≥ 0.999 for a single species or subspecies cluster were retained for diagnostic SNP locus discovery. The same framework was applied within RBT to define CRT and IRT subspecies reference groups (see Supplementary Methods).

### 2.6 Species‐ and Subspecies‐Diagnostic Loci

Genome‐wide allele frequencies were estimated separately for each reference group using VCFtools. To account for unequal sample sizes, groups were standardized by randomized subsampling prior to frequency estimation (see Supplementary Methods). Diagnostic SNP loci were defined using absolute allele frequency differences between taxa. After evaluating thresholds between 0.95 and 0.99, we selected ≥ 0.975 for species comparisons as a balance between minimizing inclusion of ancestral polymorphisms and retaining power to detect small introgressed tracts (Racimo et al. 2015). For IRT–CRT subspecies comparisons, genome‐wide divergence was low, and relaxed thresholds primarily added dispersed loci rather than contiguous ancestry blocks. We therefore adopted a more stringent cutoff (freq ≥ 0.99) for downstream analyses.

### 2.7 Chromosomal Inversion‐Diagnostic Loci

Putative interspecific inversions were explored using PCA restricted to inversion coordinates (Table 1). Patterns consistent with inversions—strong segregation into homokaryotypic clusters with heterokaryotypic intermediates, consistent with expectations for inversion polymorphisms (Knief et al. 2024)—were used as evidence that inversion regions had been correctly identified. Random genomic regions of similar size did not exhibit this pattern, instead showing patterns similar to overall population structure. Most inversions yielded the expected pattern; however, Chr13 appeared variable in all taxa and was excluded from further analyses. Inversion‐type diagnostic SNP loci were defined as sites fixed (freq = 1.0) between homokaryotypic reference groups within the remaining inversion regions (Table S1). Polarities of Chr20 and Chr22 were consistent with previous studies (Ostberg et al. 2013; Sutherland et al. 2016), and all others were inferred (Table 1). Intraspecific inversions within RBT (Pearse et al. 2019; Hale et al. 2024; Ali et al. 2025, 2026) were evaluated similarly. Individual inversion genotypes were assigned based on the normalized proportion of inversion‐type diagnostic alleles across inversion regions. Full details, including limitations regarding the intraspecific RBT chromosome 20 (Omy20) inversion overlapping with Chr20, are provided in Supplementary Methods and Results.

**TABLE 1:** Genomic locations, lengths, and orientations of chromosomal inversions across study taxa. Start and end coordinates are mapped to the Arlee rainbow trout reference genome. Taxa include interior redband trout (IRT), coastal rainbow trout (CRT), westslope cutthroat trout (WCT), and Yellowstone cutthroat trout (YCT). The chromosome 5 double inversion complex is partitioned into two distinct regions (Chr05_Inv1 and Chr05_Inv2). Inversion orientations are classified as ancestral (A), rearranged/derived (R), or variable/not taxon informative (V). Putative or unconfirmed arrangements are denoted with a question mark (?).

Inversion	Chromosome	Start (bp)	End (bp)	Length (Mb)	IRT	CRT	WCT	YCT
Chr05_Inv1	5	30,102,000	60,368,999	30.267	R	V	A	A
Chr05_Inv2	5	60,369,000	87,502,000	27.133	R	V	A	A
Chr13	13	25,743,000	28,590,000	2.847	V	V	V	V
Chr17	17	36,050,000	43,906,000	7.856	A (?)	A (?)	R	R
Chr20	20	4,356,000	21,129,000	16.773	R	R	A	A
Chr22	22	7,308,000	22,170,000	14.862	R	R	A	A
Chr29	29	2,314,000	14,206,000	11.892	A	A	R	R

### 2.8 Global Ancestry Inference

We estimated individual global ancestry proportions for each taxon using species‐ and subspecies‐diagnostic loci, recoding alleles by diagnostic group, and calculating the proportion of alleles from each group/taxon. Proportions were normalized so that ancestry components summed to one both within *O. mykiss* and across all three species groups, producing comparable *Q* values for RBT, WCT, YCT, IRT, and CRT.

We used supervised ADMIXTURE to generate another estimate of global ancestry. Reference groups for this analysis were based on individuals identified as *Q* ≥ 0.999 IRT, WCT, or YCT using normalized diagnostic locus ancestry proportions and presence in reference groups used for diagnostic discovery. We also required individuals to have all inversion genotypes consistent with their taxon based on inversion‐specific diagnostic loci. Reference CRT were chosen using high CRT ancestry proportions (*Q* ≥ 0.98) based on diagnostic loci, information on known hatchery origin, and having all inversion genotypes consistent with RBT. From these reference groups, equal samples of 55 individuals per taxon—equivalent to the number of the smallest reference group (YCT)—were chosen at random to minimize potential bias associated with uneven sample size (Meirmans 2019). All samples not chosen as reference samples were treated as potentially admixed. ADMIXTURE was run at *K* = 4 for 100 replicates with a random seed for each replicate. Variability among replicates was negligible (mean SD = 1.12 × 10^−6^ across *K* = 4 components), so ancestry proportions were averaged across all replicates to yield global ancestry proportions. Total RBT ancestry was calculated as the sum of CRT and IRT subspecies components.

### 2.9 Local Ancestry Inference

Local ancestry inference was used to generate per‐locus ancestry estimates and to visualize hybrid ancestry across collections. We used Efficient Local Ancestry Inference (ELAI) v1.01 (Guan 2014) because it can handle unphased data, parse multiple ancestry groups, is robust to uneven reference group sizes, and does not require prior knowledge about the recombination landscape (Zhou et al. 2016). For IRT, WCT, and YCT, we used the same reference groups of 55 individuals employed in ADMIXTURE. Testing showed that ELAI overestimated IRT ancestry on chromosome 5 if the CRT reference group was not split by Chr05 complex genotype since IRT are fixed for the derived form of the inversion and CRT are variable (see Results). Accordingly, two CRT reference groups were used: 31 individuals homozygous for the derived form (RBT_RR_) and 55 individuals homozygous for the ancestral form (RBT_AA_) of the Chr05 complex. Heterozygous CRT individuals (RBT_AR_) and all other samples not used as references were treated as potentially admixed. Unphased input files in bimbam format (Guan and Stephens 2008) were generated for each group using a custom R script.

We ran ELAI for each chromosome, specifying five upper clusters (−C = 5), 20 lower clusters (−c = 20), 20 EM steps (−s = 20), and a random seed. To account for uncertainty in the number of generations since admixture, ELAI was run for five different values of mixing generations (−mg = 10, 20, 30, 40, 50) and results were averaged across runs as recommended in Guan (2014). Values were chosen to encompass the likely range of generations non‐native taxa have been on the landscape. Per‐locus ancestry dosages were collapsed by chromosome and weighted by chromosome length to calculate comparable *Q* values for each individual. CRT ancestry was calculated as the sum of both CRT ancestry components and total RBT ancestry was calculated as the sum of the CRT and IRT subspecies components.

### 2.10 Hybrid Class Inference

Initially, individuals were classified as putative F1s if they exhibited approximately 50% RBT ancestry (range: 49.66%–56.22%), 50% YCT or WCT ancestry (range: 43.78%–50.34%), and the interspecific heterokaryotype at all inversions. Ancestry proportions were required to be consistent across ancestry estimation methods. Backcrosses were defined as having > 1% minor parent ancestry (the species that contributed less ancestry to a hybrid; Schumer et al. 2018). Additionally, hybrid classes strictly between RBT and WCT (excluding individuals with > 0.1% YCT ancestry) were corroborated by comparing the proportion of RBT ancestry to interspecific heterozygosity using RBT‐diagnostic loci (frequency ≥ 0.99 in RBT). Individuals were categorized into unadmixed, F1, F2, directional backcross, or complex admixture classes, with complex admixture including all remaining admixed individuals falling outside the defined F1, F2, and directional backcross boundaries. To prevent the misclassification of later‐generation hybrids, directional backcrosses were constrained by mathematical bounds adapted from Rosenthal et al. (2022). Full classification criteria are detailed in the Supplementary Methods.

### 2.11 Recombination Suppression

Recombination is suppressed in some chromosomal regions of RBT × cutthroat trout hybrids, particularly where interspecific inversions occur (Ostberg et al. 2013). If recombination is reduced within an inversion region, RBT‐diagnostic alleles located inside the inversion should be less likely to introgress into the alternate genomic background relative to loci in collinear regions of the same chromosome. Consequently, we expected higher interspecific heterozygosity (the state of carrying alleles from both parental species at a single locus) at RBT‐diagnostic loci within inversion regions compared to RBT‐diagnostic loci outside the inversion.

To test this prediction, we quantified per‐individual interspecific heterozygosity at RBT‐diagnostic loci (frequency ≥ 0.99 in RBT) located within interspecific inversion regions oppositely fixed between RBT and cutthroat and compared it to heterozygosity for loci outside the inversion on the same chromosome. Among individuals heterokaryotypic for the inversion, inversion‐specific heterozygosity was evaluated relative to the collinear portion of the chromosome to determine whether recombination suppression was localized to the inversion or extended across the entire chromosome.

Chromosomal fusions and fissions between taxa have also been documented. RBT chromosomes 20 (Omy20) and 28 (Omy28) are fused in YCT and WCT (20/28 fusion) relative to RBT (Ostberg et al. 2013; Flores et al. 2025). Interspecific heterozygosity at RBT‐diagnostic loci was compared between Omy20 and Omy28 to evaluate whether recombination suppression extended across fused chromosomes in hybrids. For fusions present in RBT relative to WCT (Omy08, Omy11, Omy15; Figure S1), fusion breakpoints were identified in the Arlee assembly using whole‐genome alignments to WCT. Fused chromosomes were partitioned into two segments corresponding to the unfused WCT state. Heterozygosity was compared between arms to determine whether Robertsonian heterozygosity—where two acrocentric chromosomes form one parent pair while a single metacentric chromosome forms the other (Fedyk and Chętnicki 2010)—influenced recombination patterns.

### 2.12 Sliding Window *F*_ST_ Analysis

To further characterize differences between reference groups and divergence across genomic regions associated with inversions, we calculated pairwise population differentiation (*F*_ST_) using the *–fst‐window‐size* and *–fst‐window‐step* parameters in VCFtools. Inversion‐aware reference groups, matching those used for ELAI, were subset to equal sample sizes (*N =* 31) and Weir and Cockerham *F*_ST_ estimates were calculated in non‐overlapping 500‐kb windows for each taxon pair. Results were partitioned into three regions: the Chr05 complex, the non‐inverted background of chromosome 5, and the remaining genome. Significance of elevated differentiation within the inversion was assessed using one‐sided Wilcoxon rank sum tests (Kolaczkowski et al. 2011), with effect sizes quantified using Cliff's delta (Meissel and Yao 2024).

### 2.13 Analytical Software

ChatGPT (OpenAI; GPT‐4o and subsequent GPT‐5‐series free‐tier models; accessed August 2024–July 2026) and Gemini (Google; Gemini 3‐series Pro and Flash models through Google AI Pro; accessed December 2025–June 2026) were used for coding assistance when writing, debugging, and troubleshooting custom shell and R scripts used in the Methods and Supplementary Methods, including scripts for figure generation. All LLM‐assisted code was reviewed, tested, and revised by the authors, and scripts were validated through execution on the study datasets, inspection of outputs, and comparison with expected behaviour and relevant software documentation; LLMs were not used to independently perform or interpret analyses. No human‐subject data were involved. LLM inputs were limited to code, error messages, and project‐specific information necessary for coding and troubleshooting.

## 3 Results

We obtained high‐quality data for 3489 fish, consisting of 2894 wild *Oncorhynchus* from the Kootenai Basin, 137 hatchery CRT, 56 hatchery IRT, 60 putative YCT, and 402 *Oncorhynchus* from Alberta hatchery lines and the Athabasca River system. All samples were genotyped at 189,984 unique biallelic SNPs (Figure S2a) with a mean depth per locus of 60.1× ±27.0 (range 20.9–436.9) and mean site missingness of 0.042 ± 0.043. From this dataset, we identified 1762 RBT, 418 WCT, and 51 YCT as initial references for species‐diagnostic locus discovery. We also identified 933 IRT and 130 CRT as references for subspecies‐diagnostic loci discovery. Thousands of species‐diagnostic loci and hundreds of subspecies‐diagnostic loci were recovered (Table S1; Figure S2b–f). Individuals used for references changed slightly based on diagnostic locus *Q*‐values, as noted above (e.g., 51 vs. 55 reference YCT). Using numerically even subsets of the reference groups and all SNPs, global genome‐wide divergence between IRT and CRT (weighted *F*_ST_ = 0.462–0.474) was significantly lower than between species (weighted *F*_ST_ = 0.843–0.902). Few loci were completely fixed between IRT and CRT outside of those within and around the Chr05 inversion complex (Figure S3).

### 3.1 Genome Structure of IRT

The IRT *de novo* consensus genetic map consisted of 11,333 SNPs (Table S2). All maps revealed that the genome structure of Kootenai IRT mirrored the Swanson RBT reference genome and all three IRT populations examined had a haploid karyotype of 29 chromosomes. This observation is in line with previous karyotype observations for IRT from the Snake and Clearwater Rivers as well as other parts of the Columbia River (Thorgaard 1983), suggesting this arrangement is consistent within the broader range of IRT. Alignment to the Swanson and Arlee RBT reference genomes showed that Kootenai IRT were homozygous for the derived form (RBT_RR_) of the Chr05 inversion complex (Pearse et al. 2019). Like Arlee and Swanson, Kootenai IRT possessed the derived form of the Omy20 intraspecific inversion found in northern populations of RBT (Hale et al. 2024). Maps showed no clear evidence of the intraspecific chromosome 26 inversion recently described in the Swanson assembly (Ali et al. 2025) in any population. A region of reduced recombination on chromosome 17 was apparent in alignments to the Arlee and Swanson assemblies, roughly in line with an intraspecific inversion described in Ali et al. (2026). This same pattern of reduced recombination was observed when the map was aligned to the Connor Lake WCT assembly, indicating greater complexity than a single inversion. Alignment to WCT also showed interspecific inversions on chromosomes 20 (Chr20), 22 (Chr22), and 29 (Chr29) consistent with Flores et al. (2025). Alignment plots are provided in Figure S4a–c.

### 3.2 Landscape Patterns of Hybridization

**FIGURE 2:**
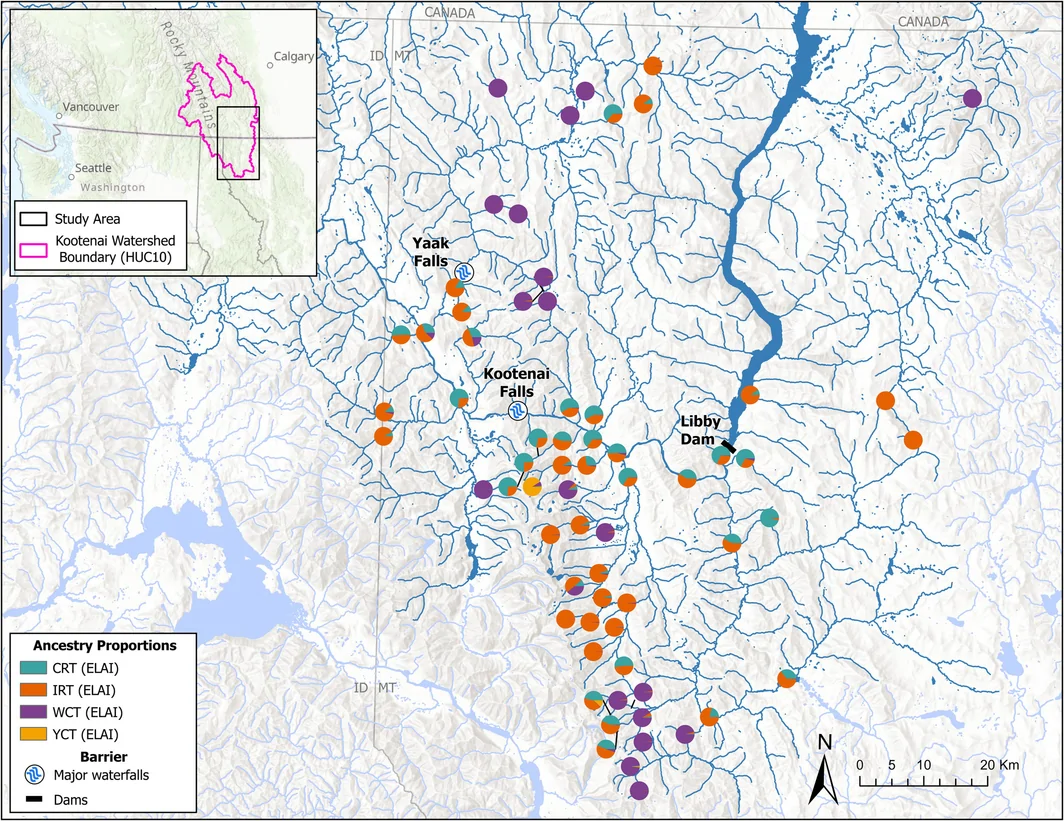
ELAI‐derived global ancestry estimates for *Oncorhynchus* collections across the Kootenai River Basin. Pie charts denote the mean proportion of coastal rainbow (CRT), interior redband (IRT), westslope cutthroat (WCT), and Yellowstone cutthroat (YCT) trout ancestry per site. Overlapping sites and temporal replicates are offset or collapsed for visibility. Sample locations within British Columbia, Canada, are not pictured.

Global ancestry estimates were highly concordant across the diagnostic locus, ADMIXTURE, and ELAI methods (Pearson's *r* > 0.99; Figure S5). We primarily used ELAI for interpreting global ancestry proportions due to its superior resolution in detecting small introgressed tracts and distinguishing minor RBT subspecies components (CRT and IRT) relative to frequency‐based approaches (see Supplementary Results for detailed comparisons). ELAI results for the Montana portion of the Kootenai drainage are visualized in Figure 2 and presented below, alongside interspecific ancestry estimates using species‐diagnostic loci. Admixture in putative IRT hatchery lineages is presented in the Supplementary Results.

**FIGURE 3:**
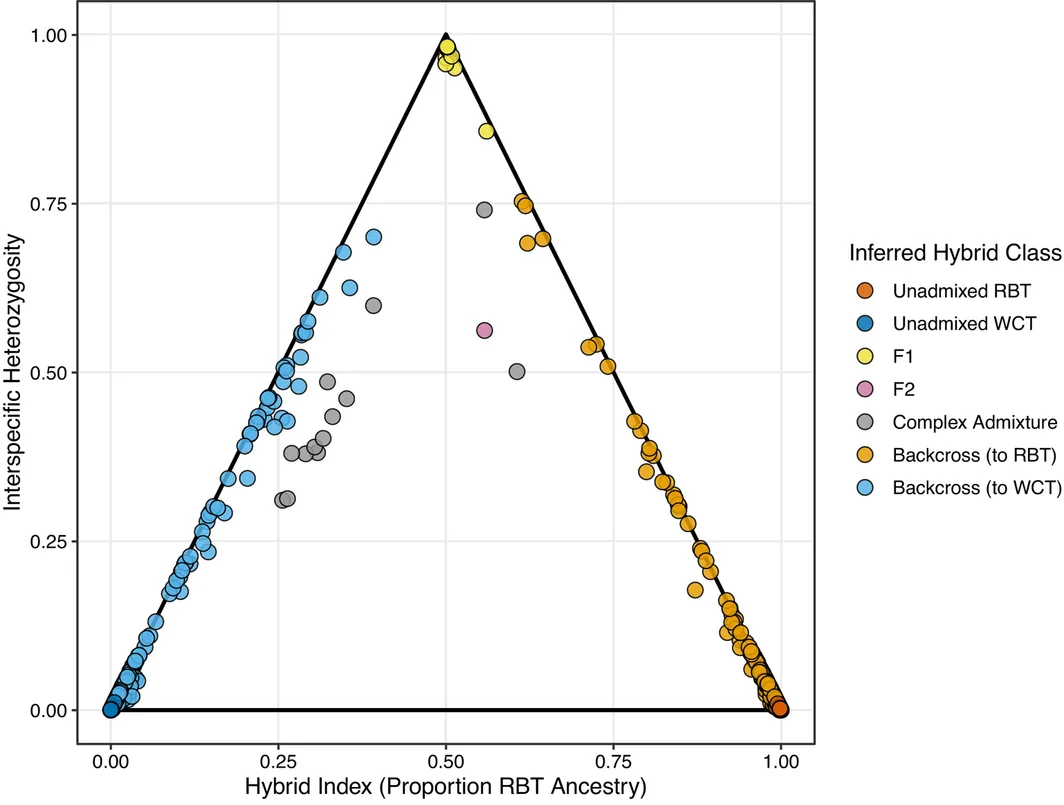
Triangle plot depicting interspecific heterozygosity versus the proportion of rainbow trout (RBT) ancestry (hybrid index) for Kootenai Basin individuals. Points are coloured by inferred hybrid class (unadmixed, F1, F2, directional backcross, or complex admixture).

Hybrids were detected in nearly all populations, though some only showed hybridization between native taxa (Figure 2). Across all collections, few true F1s were observed (*N* = 8). Additionally, F2 hybrids between RBT and WCT were nearly absent in our dataset (Figure 3). Most hybrid individuals were likely the result of extensive backcrossing, with many having less than 20% ancestry from another taxon. This was reflected in most collections having low average interspecific ancestry. Interspecific backcrossing was asymmetrical, with most complex admixture occurring in majority‐WCT individuals (Figures 3 and S6).

Intraspecific RBT hybridization was common, with introduced CRT ancestry prevalent in much of the system (Figure 2), though patterns varied across the Kootenai Basin. Mainstem Kootenai River and Fisher River samples, along with lower tributaries, showed extensive admixture with CRT and low frequencies of WCT and YCT ancestry. In the Yaak River drainage, populations above major barrier falls were primarily native WCT or IRT ancestry aside from augmented lake populations. In drainages lacking absolute barriers, such as parts of Big Cherry and Libby creeks, the proportion of CRT ancestry primarily followed a pattern of decreased occurrence with distance upstream from the mainstem. This gradient was altered in tributary systems connected to headwater lakes, where high proportions of non‐native ancestry were detected upstream of less‐admixed tributary populations. Detailed drainage‐specific ancestry estimates at the population and individual level can be found in Tables S3a–S3d.

While the broader application of inversions for lineage identification depends on confirming they are truly diagnostic, intraspecific inversions between IRT and CRT on Chr05 were useful for validating CRT ancestry in majority‐IRT populations and in interspecific hybrid zones. The Omy20 intraspecific inversion was less useful, with ancestral homozygotes and heterozygotes observed very rarely across the dataset (*N* = 3 and 25 respectively), primarily in two lakes and the mainstem Kootenai River (additional information in Supplementary Results; Tables S3a–S3d). Variants of the Chr05 complex were much more widespread and informative. Because the Chr05 complex is fixed for the RBT_RR_ form in IRT and variable in CRT, the presence of RBT_AR_ or RBT_AA_ genotypes served as an informative marker for CRT admixture. For example, collections from lower Bear Creek (Bear_20; Bear_21) in the Libby Creek drainage had average global CRT ancestry of 3.80% and 4.66%, respectively. CRT admixture was estimated to be distributed across the majority of the genome in these populations (Figure S7) and was present in all sampled individuals across both collections. The presence of the RBT_AR_ genotype in 5.2% (*N* = 5) and 9.5% (*N* = 9) of those individuals, respectively, confirmed that these low ancestry estimates represent true non‐native introgression, rather than statistical noise or methodological artefacts associated with resolving small amounts of admixture.

### 3.3 Recombination Suppression Associated With Rearrangements

**FIGURE 4:**
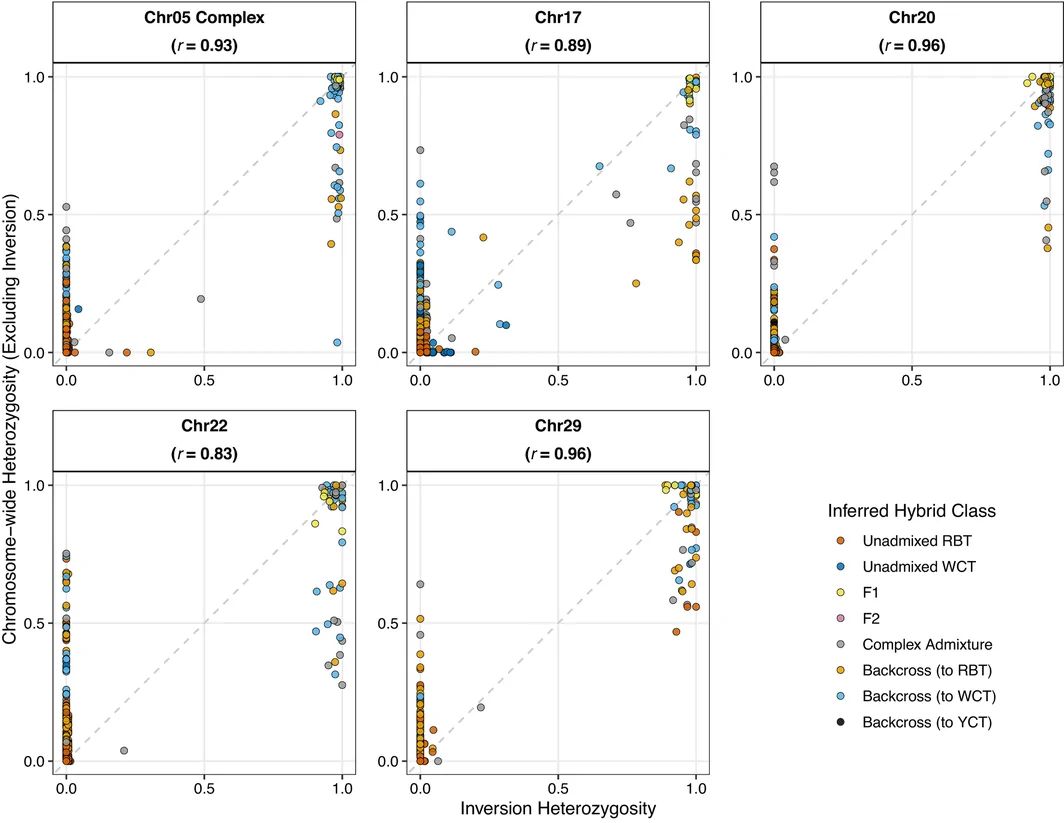
Relationship between inversion‐specific heterozygosity and chromosome‐wide heterozygosity (excluding the inversion) at RBT‐diagnostic loci. Panels display known interspecific inversions (Table 1). Points are coloured by inferred hybrid class and Pearson correlation coefficients (*r*) are shown for each structural variant.

Inversion genotype was typically sufficient to predict heterozygosity across entire chromosomes, with heterozygosity patterns inside inversion regions closely matching those across the rest of the chromosome. For inversions on Chr05, Chr20, and Chr29, recombination in heterokaryotypic hybrids was effectively absent; interspecific heterokaryotypic hybrids had high heterozygosity across the entire chromosome while homozygotes showed the opposite pattern (Pearson's *r* > 0.92; Figure 4). The Chr22 inversion exhibited similar patterns of suppression with few recombinants observed, but the weaker relationship between inversion and genome‐wide heterozygosity (*r* = 0.83) could indicate that inversion boundaries were not cleanly defined. The Chr17 inversion exhibited strong evidence of recombination suppression (*r* = 0.89), though recombinant genotypes were observed, indicating that suppression is not as complete (Figure 4). These patterns held for some backcrosses with < 20% hybrid ancestry, suggesting that suppression likely persists despite backcrossing.

The genotype of the Chr20 inversion, and thus interspecific heterozygosity along Omy20, was highly correlated with interspecific heterozygosity across the entirety of Omy28 (*r* = 0.98; Figure S8). Hybrids were likely Robertsonian heterozygotes carrying two RBT chromosomes (Omy20 and Omy28) and one copy of the 20/28 fusion. Co‐suppression across both RBT chromosomes associated with the inversion suggests that both inversion and Robertsonian heterozygosity contribute to strong recombination suppression in interspecific hybrids. Similarly, in chromosomes associated with fusions present in RBT relative to WCT, heterozygosity across Arm 1 was strongly associated with heterozygosity across Arm 2 (*r* ≥ 0.93; Figure S9), suggesting that Robertsonian heterozygosity contributes to recombination suppression as seen in RBT × YCT hybrids (Ostberg et al. 2013). Despite the strength of these relationships, recombinants were observed much more frequently for these chromosomes than for inversions and interchromosomal rearrangements, indicating that Robertsonian heterozygosity more weakly suppresses recombination. Again, both inter‐ and intra‐chromosomal patterns held for late‐stage backcrosses.

### 3.4 Intraspecific Inversion Genotype Distribution and Potential Ecological Associations

The geographic distribution of Chr05 genotypes in majority‐RBT individuals revealed apparent stratification in the distribution of RBT_AR_ and RBT_AA_ genotypes based on location in each drainage. When collections were categorized by habitat type (mainstem river, valley floor, lake, reservoir, or tributary stream), RBT_AA_ individuals were only present in habitats associated with life histories dependent on rearing in larger streams (fluvial fish) or lakes (lacustrine fish). Higher‐gradient tributary streams, associated with resident or fluvial fish, completely lacked RBT_AA_ individuals. Weighted means indicated that tributary sites contained no RBT_AA_ individuals and had the highest frequency of RBT_RR_ (97.8%) individuals. In contrast, RBT_AA_ genotypes were present in all non‐tributary habitats, occurring at low frequency in reservoir‐derived collections (1.16%) and at higher frequencies in collections from lakes, rivers, and valley‐floor sites (range: 10.00%–11.86%; Table 2). Twenty RBT_AA_ individuals were primarily IRT × CRT hybrids but carried cutthroat trout ancestry in regions other than Chr05 (0.055% to as much as 20.65%), indicating that backcrossing in multitaxon hybrid zones or swarms can result in hybrids with RBT‐only Chr05 genotypes that still follow the same pattern of genotype‐associated habitat stratification.

**TABLE 2:** Weighted mean frequencies of rainbow trout (RBT) chromosome 5 (Chr05) inversion complex genotypes across habitat types. Genotypes include homozygous ancestral (RBT_AA_), heterozygous (RBT_AR_), and homozygous rearranged (RBT_RR_). Frequencies represent Kootenai Basin collections binned by habitat type containing individuals with majority‐RBT ancestry at the Chr05 complex, weighted by sample size (*N*).

Habitat type	RBT_AA_	RBT_AR_	RBT_RR_	*N*
Tributary	0.00	0.02	0.98	1649
Valley Floor	0.12	0.29	0.59	59
River	0.10	0.31	0.59	100
Lake	0.11	0.16	0.73	231
Reservoir	0.01	0.13	0.86	86

### 3.5 Potential Selection Against Interspecific Heterokaryotypic Inversions

Patterns of reduced WCT ancestry within interspecific inversions were observed in IRT populations isolated from non‐native introgression. We examined two naturally introgressed IRT populations that contain no definitive CRT ancestry but differ in connectivity to evaluate whether heterokaryotypes are observed in the absence of ongoing gene flow. In the upper East Fork Yaak drainage (EF_Yaak_20, EF_Yaak_21, EF_Yaak_22, EF_Yaak_24; *N* = 430), where hybridization between native taxa is rare but ongoing (Leary et al. 2008), heterokaryotypic individuals were detected at low frequency (4.65%, *N* = 20). Each of these individuals carried between one and three heterokaryotypic inversions across the five inversion regions fixed between IRT and WCT (Table 1), including two individuals with > 99% inferred IRT ancestry. In contrast, in upper Bear Creek (Libby Creek drainage; Bear_17, Up_Bear_22, Bear_24; *N* = 216), where IRT are the only taxon present above a barrier and contemporary introgression from WCT is unlikely, no heterokaryotypic inversions were detected despite 90.23% of individuals exhibiting trace WCT ancestry (0.1%–1.24%) in colinear genomic regions. In collections from lower Bear Creek below the barrier (*N* = 191), a single heterokaryotypic inversion was detected (*N* = 1), confirming interspecific heterokaryotypes can occur when low levels of gene flow are maintained.

**FIGURE 5:**
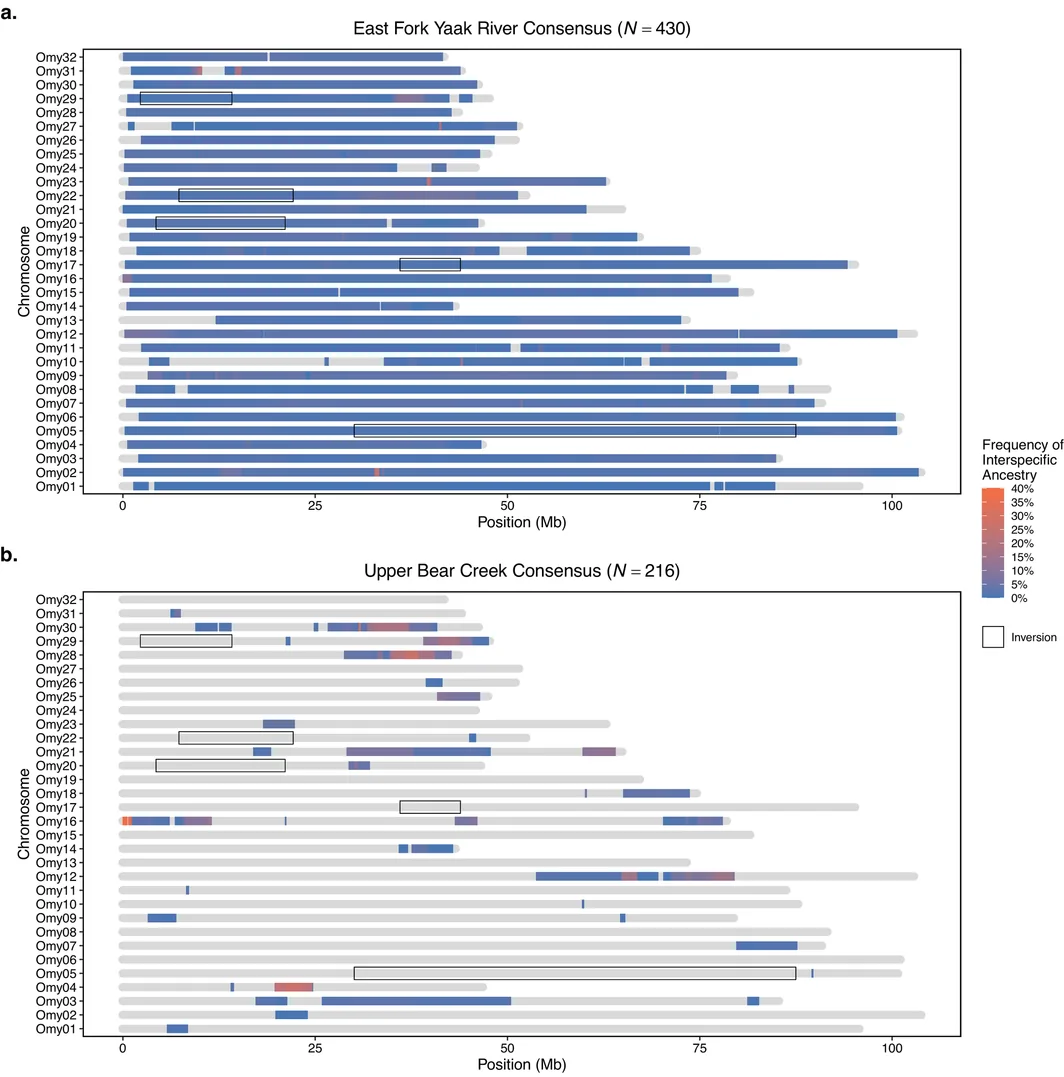
Consensus plots showing the frequency of interspecific ancestry across the genome for two Kootenai Basin collections: (a) East Fork Yaak River and (b) Upper Bear Creek. Both populations are primarily interior redband trout (IRT) major parent ancestry (not shown). As alignment to the Arlee rainbow trout reference genome was performed, chromosome names match that nomenclature. Heatmaps display the distribution and population‐level frequency of homozygous westslope cutthroat trout (WCT) and heterozygous (IRT/WCT) hybrid ancestry tracts derived from ELAI and calculated in 5‐kb non‐overlapping windows on each chromosome. The colour gradient indicates the frequency of interspecific ancestry in each genomic window across individuals. Light grey horizontal bars show the full physical extent of each chromosome and serve as the background onto which interspecific ancestry frequencies are plotted; grey regions indicate windows with no detectable interspecific ancestry. Fixed inversions between IRT and WCT are denoted by black rectangles.

This difference between populations is also evident in genome‐wide ancestry patterns. Visualization of the frequency of hybrid ancestry blocks identified by ELAI across all East Fork Yaak individuals showed large, low‐frequency heterozygous blocks across the population, characteristic of recent backcross hybrids (Figures 5 and S10a). Across the East Fork Yaak collections, 11 individuals had greater than 10% WCT ancestry (11.05%–21.25%), while the remainder were predominantly RBT (> 90%). By contrast, the upper Bear population showed highly fragmented heterozygous ancestry blocks, consistent with historical introgression followed by loss of hybrid ancestry due to selection and/or drift (Figures 5 and S10b). Trace WCT ancestry in upper Bear individuals was scattered across collinear regions, and some genomic regions showed increased frequency of hybrid ancestry, but interspecific inversion regions were devoid of WCT ancestry.

### 3.6 Potential Interspecific Incompatibilities With the Chr05 Complex

Across admixed collections, the prevalence of heterokaryotypic inversions generally scaled with genome‐wide ancestry (Figure S11). The Chr05 complex is structurally distinct from the other examined inversions; while most inversions are differentially fixed between RBT and cutthroat trout taxa, the Chr05 complex remains polymorphic in CRT, maintaining both colinear (RBT_A_) and inverted (RBT_R_) forms. Because the RBT_A_ haplotype is colinear with the ancestrally oriented WCT (WCT_A_) and YCT (YCT_A_) haplotypes, we initially hypothesized increased recombination in interspecific hybrids with CRT, leading to more prevalent introgression across chromosome 5. However, our data revealed the opposite pattern, indicating a potential incompatibility specifically between the WCT_A_ and RBT_A_ haplotypes.

**FIGURE 6:**
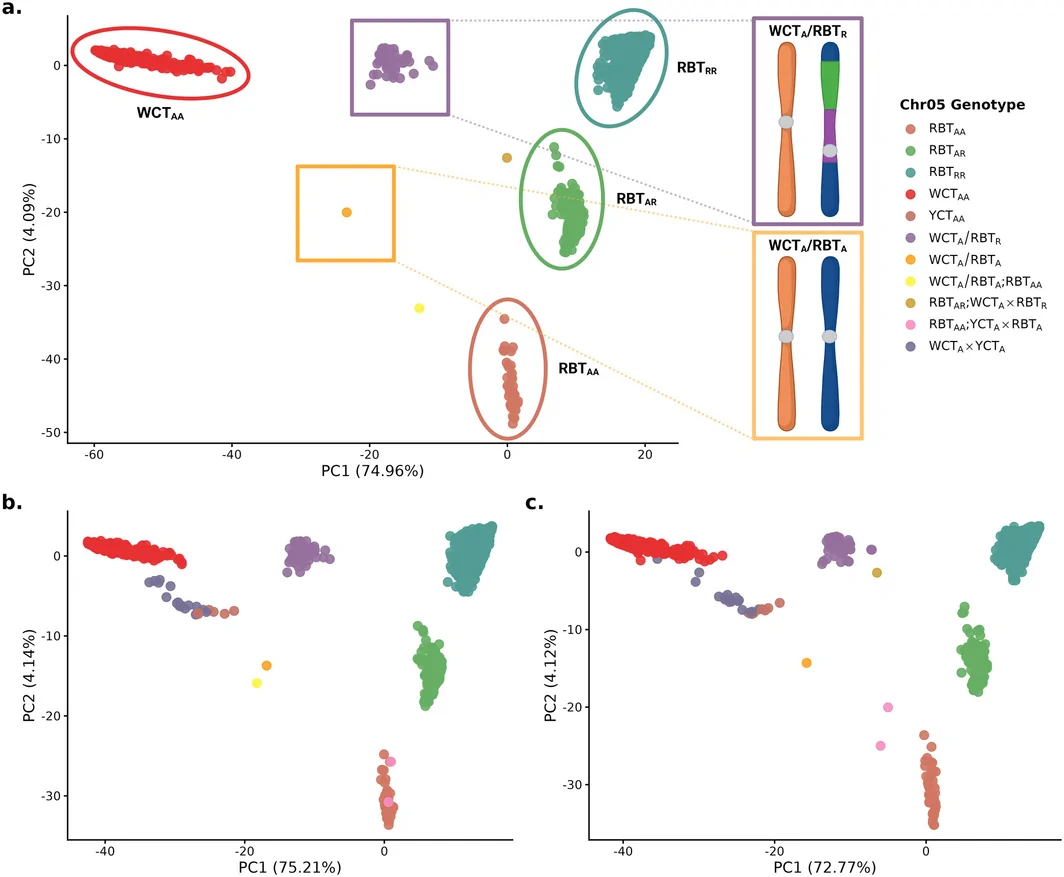
Principal component analysis of genotypic variation within the chromosome 5 (Chr05) double inversion complex. Panels display clustering based on SNPs within (a) the full inversion complex (excluding Yellowstone cutthroat trout [YCT] and YCT recombinants), (b) inversion 1, and (c) inversion 2. Genotype labels indicate haplotype composition, with subscripts denoting ancestral (A) or rearranged (R) arrangements for westslope cutthroat trout (WCT) and rainbow trout (RBT) haplotypes (e.g., WCT_A_/RBT_R_). Recombinant genotypes within inversions are denoted with a cross (×). For partial recombinants affecting only one inversion, genotypes are separated by a semicolon in order by inversion (e.g., WCT_A_/RBT_A_; RBT_AA_).

We performed PCA using SNPs across the Chr05 complex and its individual inversions for all Kootenai Basin samples (Figure 6). Position in PCA space and inversion diagnostic locus proportions were used to diagnose whether hybrids were the product of RBT_A_ or RBT_R_ pairings with cutthroat taxa or pairings of the two cutthroat taxa (Table S4). Of the observed Chr05 hybrids (*N* = 83), 58 were WCT_A_/RBT_R_ and 20 were WCT_A_ × YCT_A_. All observed RBT × WCT F1 individuals (*N* = 8) had WCT_A_/RBT_R_ genotypes and were found in habitats associated with migratory behaviour. Offspring resulting from a non‐recombined colinear RBT_A_ and WCT_A_ pairing (WCT_A_/RBT_A_) were exceedingly rare, with only one interspecific WCT_A_/RBT_A_ individual detected among 63 RBT × cutthroat trout hybrids (1.59%). This individual occurred in Flower Creek, a WCT‐majority ancestry population. It was predominantly WCT in ancestry (75.36%) and was heterokaryotypic at all other inversions, representing a backcross between an F1 RBT × WCT hybrid and WCT.

Partial recombinants between RBT_A_ and WCT_A_ or YCT_A_ were detected at low frequency, with one individual that was heterokaryotypic only across Chr05_Inv1 (WCT_A_/RBT_A_; RBT_AA_) and two individuals that were interspecific recombinants with YCT only across Chr05_Inv2 (RBT_AA_; YCT_A_ × RBT_A_). Another individual had an RBT_AR_ genotype across Chr05_Inv1 and was a partial interspecific recombinant across Chr05_Inv2 (RBT_AR_; WCT_A_ × RBT_R_). All partial recombinants between RBT and the cutthroat taxa were collected in lakes and the mainstem Kootenai River or its direct tributaries. None of those individuals were heterokaryotypes at any other inversions and all were majority RBT ancestry (98.12%–98.95%), indicating that portions of cutthroat Chr05 can remain as minor parent ancestry in complex RBT backcrosses.

Taken together, the presence of only a single WCT_A_/RBT_A_ individual and few recombinants over decades of sampling hints at the possibility of a colinear haplotype pairing being associated with an incompatibility between RBT and WCT, and the possibility that individuals with this genotype are heavily selected against, even at the F1 stage. The complete lack of non‐recombinant YCT_A_/RBT_A_ individuals could indicate this same pattern in hybrids between RBT and YCT or other cutthroat trout species.

### 3.7 Genomic Divergence Associated With the Chr05 Complex

**FIGURE 7:**
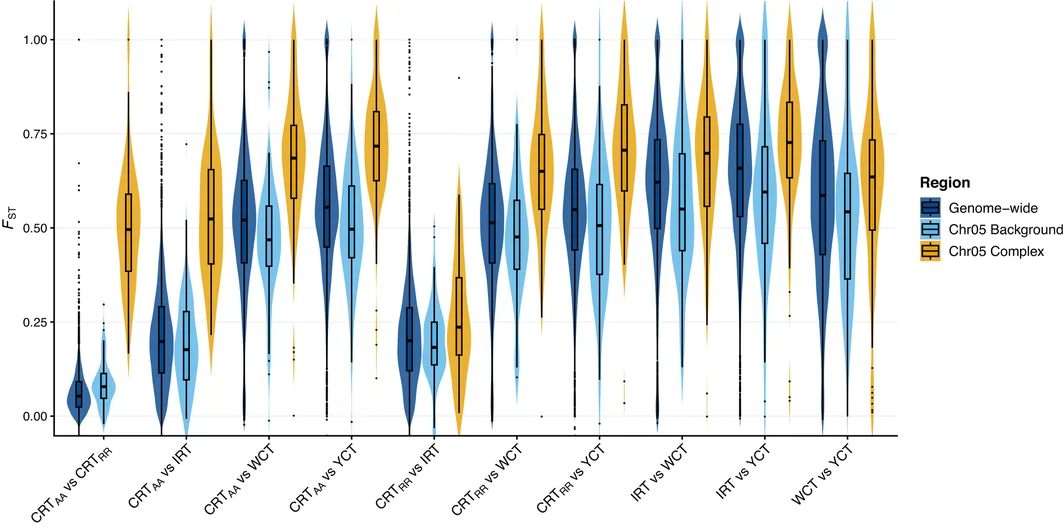
Pairwise genomic differentiation (*F*_ST_) within the chromosome 5 (Chr05) double inversion region (Chr05 Complex) compared to the remainder of chromosome 5 (Chr05 Background) and the genome‐wide background (excluding the Chr05 Complex). Comparisons include coastal rainbow trout (CRT) carrying homozygous ancestral (CRT_AA_) or derived (CRT_RR_) Chr05 genotypes, interior redband trout (IRT), westslope cutthroat trout (WCT), and Yellowstone cutthroat trout (YCT).

Median *F*_ST_ within the Chr05 complex was significantly elevated in all RBT and cutthroat comparisons, including comparisons between taxa sharing the same chromosomal orientation (Wilcoxon rank sum: *p* < 0.001; Table S5; Figure 7). This region was also distinct relative to the genome‐wide average and the collinear sections of chromosome 5 outside of the inversion region. CRT_AA_ compared to WCT and YCT exhibited median inversion *F*_ST_ values of 0.685 and 0.717, respectively. These values were substantially higher than their non‐inverted chromosome 5 backgrounds (Cliff's delta = 0.648 and 0.656, respectively) as well as their genome‐wide averages (Cliff's delta = 0.496 and 0.524, respectively). Divergence was similarly elevated in CRT_RR_ and IRT comparisons to the cutthroat taxa (Table S5). Even between cutthroat taxa, divergence in the Chr05 complex was higher than the genome‐wide background (median 0.635 vs. 0.586; *p* = 0.044), though the effect size of this difference was minor (Cliff's delta = 0.097). Overall, the extreme divergence of the collinear RBT_AA_ and cutthroat genotypes highlights that high differentiation is not strictly due to inversion orientation and may be due to divergence via selection.

## 4 Discussion

Here, we examine how introductions of non‐native trout have disrupted hybrid zones between native IRT and WCT, shifting spatially limited hybridization toward geographically expanding, multispecies admixture and hybrid swarm formation, primarily through the introduction of CRT. Our results suggest that inversion polymorphisms carried by introduced CRT contribute to this transition by influencing dispersal, recombination, and the persistence of hybrid ancestry. In particular, the Chr05 inversion complex provides a potential mechanism linking introduced CRT ancestry to increased migration and straying, while inversions fixed between RBT and WCT suppress recombination and appear to constrain which introgressed genomic regions persist through subsequent generations. Together, these processes provide a framework for understanding how introduced structural variation can alter both the geographic extent and genomic composition of native hybrid zones.

### 4.1 The Role of Migration in Landscape Patterns of Hybridization

The contrast between populations with and without CRT ancestry illustrates this disruption at the landscape scale. Where CRT were absent, IRT and WCT hybrids were limited to backcrosses with IRT and did not result in hybrid swarm formation—defined as populations in which non‐native ancestry exceeds 90% of individuals and spans more than half of the genome (Kovach et al. 2025). In contrast, hybridization involving CRT was strongly associated with extensive admixture and hybrid swarm formation, especially in headwater lakes or tributaries with a history of CRT stocking and direct connectivity to larger river corridors (Figure 2). These patterns suggest that CRT‐introgressed individuals disperse broadly through connected river networks but exhibit higher reproductive success in lower tributary reaches or where upstream movement is constrained by barriers, producing the spatial gradients in introgression observed here and in other systems (Dangora et al. 2023). Altogether, the spread of non‐native ancestry and the collapse of spatially restricted hybrid zones into hybrid swarms appear closely tied to the movement of CRT‐introgressed individuals, indicating that differences in dispersal, combined with variation in mating behaviour such as spawn timing and habitat choice, are central to understanding why introgression expands dramatically in some drainages but remains limited in others.

### 4.2 Chr05 Habitat Stratification and Migratory Propensity

One prominent mechanistic candidate for the genetic basis of migration and straying in this hybrid zone is the Chr05 complex, which has been linked to migratory behaviour in RBT and exhibits striking habitat‐associated stratification in our data. In natural CRT populations, the RBT_AA_ and RBT_AR_ genotypes are associated with increased migratory propensity relative to RBT_RR_ individuals, with effects that are sex‐biased toward females (Pearse et al. 2019; Rundio et al. 2021; Kobayashi et al. 2024). Although we did not examine sex‐based differences, we found that individuals carrying the RBT_AA_ genotype—including some interspecific hybrids—were detected exclusively in habitats associated with migratory life histories, and RBT_AR_ individuals were far more common in these habitats than in low‐productivity tributary streams (Table 2).

These patterns mirror observations by Arostegui et al. (2019), who found that landlocked RBT populations retain the RBT_A_ haplotype and that this haplotype is associated with lacustrine rather than tributary environments. The absence of RBT_AA_ and deficiency of RBT_AR_ individuals in tributary streams leads us to hypothesize that fish with these genotypes may spawn in tributaries but that offspring are likely to out‐migrate rapidly to rear in more productive habitats, paralleling the anadromous life history of populations with ocean access. This hypothesis is supported by evidence that the RBT_A_ haplotype is also associated with slower growth and development (Nichols et al. 2008; Hecht et al. 2013; Pearse et al. 2019; Rundio et al. 2021; Beulke et al. 2023), suggesting that carriers may require greater resources than those available in tributaries, further promoting outmigration.

**FIGURE 8:**
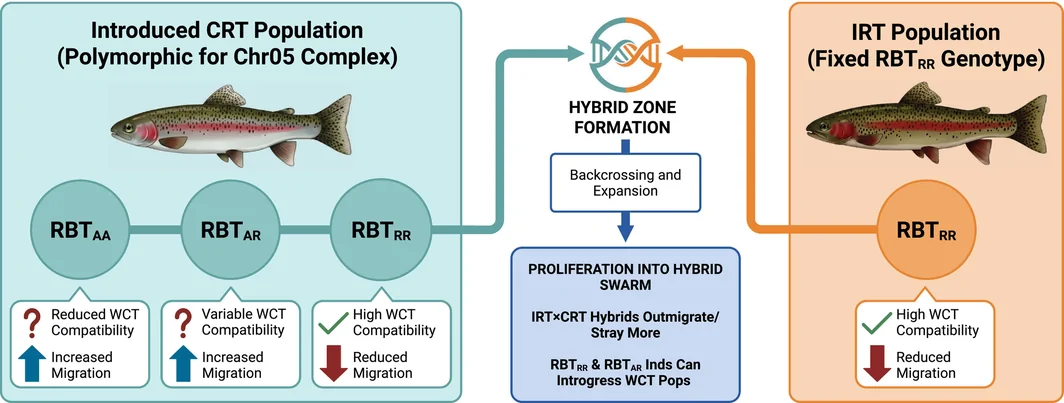
Hypothesized model of coastal rainbow trout (CRT)‐mediated hybrid swarm formation. The diagram illustrates how introduced CRT—polymorphic for the chromosome 5 (Chr05) double inversion complex (RBT_AA_, RBT_AR_, RBT_RR_)—interact with native interior redband trout (IRT; fixed for RBT_RR_). Resulting intraspecific hybrids are hypothesized to exhibit increased dispersal, facilitating contact and subsequent introgression with native westslope cutthroat trout (WCT) populations. Question marks indicate uncertainty regarding compatibility between the RBT_A_ and WCT_A_ haplotypes.

Because the RBT_A_ haplotype is naturally absent from IRT populations, hybridization between IRT and CRT introduces RBT_A_ into inland systems, potentially increasing migratory and straying behaviour among intraspecific hybrids. We propose that hybridization between IRT and CRT produces individuals that are both viable and more likely than native IRT to disperse across the landscape. This mechanism is consistent with range‐wide observations that hybridization between RBT and WCT is most extensive where the taxa are naturally sympatric (McKelvey et al. 2016), and we suspect this pattern is the consequence of historical CRT introductions into these habitats. In the Kootenai Basin, IRT populations likely act as a genetic bridge: they readily hybridize with introduced CRT and already occur in proximity to WCT, rapidly bringing intraspecific RBT hybrids into contact with WCT (Figure 8). Asymmetrical patterns of hybridization corroborate this; complex backcrossing primarily occurred in the direction of WCT (Figure 3), indicating that RBT population expansion facilitates hybrid zone expansion and subsequent introgression or displacement of WCT populations.

Additional genomic regions may also contribute to migratory behaviour. Regions homeologous to Chr05 on RBT chromosomes 1 and 12 (Omy01; Omy12) containing the *CLOCK* and *MAPK10* genes have been implicated in migratory phenotypes in RBT populations within their northern distribution in North America (Nichols et al. 2007; Hecht et al. 2013; Pearse et al. 2019). Other loci potentially associated with migratory propensity in RBT × WCT hybrids (e.g., Strait 2020) could further modify dispersal. Although these loci were not examined here, dispersal behaviour is likely polygenic, influencing the extent to which CRT‐introgressed hybrids disperse across the landscape. Still, the Chr05 complex provides a mechanistic basis for how the increased dispersal of CRT‐admixed individuals drives the expansion of hybrid swarms across the basin. At the same time, Chr05 and other interspecific inversions can constrain how ancestry is inherited and could impose fitness costs.

### 4.3 Recombination Suppression and Selection Against Heterokaryotypes

One potential constraint on interspecific introgression is recombination suppression associated with chromosomal inversions and Robertsonian rearrangements. Consistent with previous work on YCT (Ostberg et al. 2013), our results indicate that RBT, WCT, and YCT genomes generally introgress freely, but differences in chromosomal rearrangements can impede recombination across large portions of specific chromosomes in RBT × cutthroat hybrids (Figures 4, S8 and S9). Robertsonian rearrangements appear to impede recombination less frequently. Although such effects were previously documented through the F2 generation in RBT × YCT crosses (Ostberg et al. 2013), we found evidence that recombination suppression—particularly that driven by inversions—can persist well beyond this stage in natural settings despite fitness consequences.

While structural variation allows linkage blocks to persist in hybrid genomes, the resulting heterokaryotypes also carry distinct biological costs. Fitness consequences for RBT × WCT are likely stronger in natural settings. We observed few F1 individuals and a near‐absence of F2 hybrids (Figure 3), suggesting that severe reductions in survival and fertility primarily affect advanced‐generation hybrids rather than parental backcrosses, a pattern previously observed in RBT × YCT hybrids (Rosenthal et al. 2022). Inversions may help explain this and other documented fitness consequences in RBT × WCT hybrids. Fitness declines of up to 50% in WCT hybrids with at least 20% RBT ancestry (Muhlfeld, Kalinowski, McMahon, et al. 2009) and reduced fecundity (Corsi et al. 2013)—consistent with the expected fertility effects of inversions (King 1993; Wang et al. 2025)—have been reported. At the same time, greater ancestry from non‐native RBT strains is strongly associated with dispersal behaviour in RBT × WCT hybrids (Rubidge and Taylor 2004; Boyer et al. 2008; Muhlfeld, McMahon, Belcer, and Kershner 2009; Kovach et al. 2015; Strait et al. 2021), producing the paradoxical spread of hybrid ancestry despite lowered fitness (Bourret et al. 2022). Continued introgression may therefore occur primarily through repeated backcrossing of dispersing F1 hybrids into parental RBT or WCT populations rather than through persistent F2+ lineages, consistent with the lack of F2s observed here and in a previous study of RBT × WCT hybridization in the Kootenai Basin (Rubidge and Taylor 2004). Reduced growth and developmental performance may contribute to this migratory tendency. Analogous to the RBT_A_ haplotype's influence on migration in RBT (see above), fitness costs associated with interspecific inversion heterokaryotypes, combined with environmental selection against hybrids, may drive movement out of tributaries into more productive habitats for rearing. These surviving hybrids may then stray into new tributary habitats to spawn as adults, thereby spreading introgression while avoiding the formation of persistent F2+ swarms.

Empirical support for selection against interspecific heterokaryotypes is apparent when contrasting populations with different histories of admixture. Comparison of East Fork Yaak and upper Bear IRT populations suggests that recombination suppression initially maintains large blocks of hybrid ancestry, but heterokaryotypic inversions are eventually eliminated over time, particularly in the absence of continued gene flow. Across other Kootenai collections, heterokaryotypic inversions were less frequent in later‐generation backcrosses (Figure S11), suggesting this pattern may hold for anthropogenic hybrid zones. More broadly, these observations align with theoretical expectations that local recombination rates are a primary determinant of minor parent ancestry retention across the genome.

Hybrid fitness can be reduced via ecological selection, hybridization load, or negative epistatic interactions between parental genomes (Moran et al. 2021), including intrinsic fitness consequences like hybrid sterility and other incompatibilities (King 1993; Noor et al. 2001). Selection against minor parent ancestry is generally stronger in low‐recombination regions where these genetic incompatibilities or locally adaptive loci are located (Schumer et al. 2018; Moran et al. 2021). Because chromosomal inversions suppress recombination, they limit the breakdown of deleterious allele combinations during backcrossing and maintain linkage among these co‐adapted or incompatible loci (Kirkpatrick and Barton 2006; Wellenreuther and Bernatchez 2018; Wang et al. 2025). By maintaining this linkage, inversions reinforce selection against their persistence in the major parental background. Empirical studies further demonstrate that structural variants can maintain strong barriers to gene flow and reduce introgression across large genomic blocks in hybrids (Le Moan et al. 2024; Blain et al. 2025). In line with these mechanisms, the lack of hybrid ancestry within interspecific inversions in our dataset—while trace WCT ancestry is still present in collinear regions—points to locus‐specific selective processes influencing ancestry at inversions rather than genome‐wide demographic effects.

Consistent with previous observations of selection against RBT ancestry in WCT populations (Kovach et al. 2015, 2016), these results suggest that interspecific inversion regions may exhibit significantly lower levels of admixture compared to collinear regions of the genome in populations experiencing non‐native RBT × cutthroat trout hybridization when backcrossing toward WCT primarily occurs. However, because our evaluation focused on a system with a native RBT lineage (IRT), future work should explicitly test whether interspecific inversion regions are under similar negative selection in hybrid zones where RBT are entirely non‐native. Additionally, the presence of trace minor parent ancestry at increased frequency in collinear genomic regions (Figure 5) is consistent with higher recombination rates allowing fragments to escape selection against linked deleterious alleles. However, their persistence at these frequencies raises the possibility of adaptive introgression (Moran et al. 2021), though future work is needed to test whether these retained alleles provide a selective advantage rather than simply reflecting neutral introgression in regions where selection is relaxed.

### 4.4 Inversions on Chr05 May Mediate Hybrid Success

The Chr05 complex provides a striking example of how specific genetic interactions may influence interspecific hybrid swarm formation. The near‐absence of WCT_A_/RBT_A_ genotypes (Figure 6)—despite both haplotypes circulating within admixed populations—initially suggests that the RBT_A_ haplotype may be functionally incompatible with WCT_A_ genetic backgrounds, restricting successful reproduction to a subset of migrant hybrids following contact with WCT (Figure 8). Given the extensive number of Kootenai individuals genotyped in this study (*N* = 2894), we hypothesize that the underrepresentation of this combination represents a biological restriction rather than a sampling artefact.

However, several alternative explanations may contribute to the rarity of WCT_A_/RBT_A_ genotypes. Geographic and demographic segregation could limit the formation of these hybrids, as RBT_AA_ individuals are largely associated with migratory and lacustrine habitats, while WCT populations are presently (but not historically) found more commonly in headwater streams. Generalized selection against RBT_A_ haplotypes or RBT_AA_ individuals in these environments could further depress WCT_A_/RBT_A_ frequencies, regardless of intrinsic compatibility. Sampling biases resulting in the capture of fewer hybrids are also possible.

The divergence between RBT_AA_ and cutthroat genotypes is as extreme as that observed between alternative inverted arrangements (Figure 7), further hinting that Chr05 could be associated with a stronger incompatibility than seen with the other interspecific inversions. Our findings align with studies in other hybridizing species showing that genomic regions exhibiting elevated divergence between species are often enriched for loci that reduce hybrid fitness (Schumer et al. 2014) and can contribute disproportionately to barriers to gene flow in hybrid genomes (Elgvin et al. 2017). Genes near identified incompatibilities tend to be more divergent than genes located further away, suggesting that high sequence divergence can serve as a proxy for potential incompatibilities. Although the precise genetic basis remains unresolved, the pattern of the Chr05 complex is consistent with deleterious epistatic interactions among alleles within and between inversions or other loci across the genome. Such interactions are characteristic of Bateson–Dobzhansky–Muller incompatibilities (BDMIs), in which alleles that evolved independently in diverging lineages reduce fitness when combined in hybrids (Orr and Turelli 2001; Cutter 2012).

While chromosomal inversion polymorphisms are common within species, their consequences for interspecific hybridization, particularly when polymorphic in only one parental species, remain incompletely characterized. In the Kootenai, alternate Chr05 inversion haplotypes are fixed between native IRT and WCT, in line with the accumulation of fixed inversions seen in other naturally sympatric taxa (Hooper and Price 2017; Knief et al. 2024). However, invasive CRT have reintroduced the RBT_A_ haplotype, creating anthropogenically mediated polymorphism. As RBT_A_ and WCT_A_ are collinear, they can likely recombine in hybrids, disrupting co‐adapted gene complexes and exposing BDMIs, possibly contributing to the underrepresentation of RBT_A_/WCT_A_ genotypes (Figure 6). Conversely, the high frequency of RBT_R_ haplotypes in IRT promotes their persistence within IRT × CRT × WCT hybrids (Figure 8); pairing the inverted RBT_R_ with WCT_A_ suppresses recombination, reducing formation of deleterious recombinant genotypes. Recombinant genotypes were rare (Figure 6) and occurred in late‐generation hybrids with ancestry from multiple RBT and cutthroat taxa, retaining only portions of the Chr05 complex as minor‐parent ancestry and no heterokaryotypic arrangements at other inversions. This pattern suggests these combinations are infrequently produced, likely due to recombination suppression (Stathos and Fishman 2014), and face negative selection similar to other inversions. Together, these patterns indicate that Chr05‐associated incompatibility may function as a genomic filter shaping lineage persistence following secondary contact.

Sex‐specific biases in the transmission or survival of Chr05 haplotypes may further contribute to these patterns. The Chr05 complex's partial homeology with the RBT sex chromosome (Omy29) has been proposed as a mechanism underlying sex‐dependent dominance, though the exact role of sex chromosomes in resolving this conflict remains unknown (Pearse et al. 2019). Notably, the centromeric half of Omy29 (0–26.28 Mb), which shares homeology with 6.45 Mb of inversion 1 in Chr05 and contains the master sex‐determining gene in RBT (Pearse et al. 2019), also contains the Chr29 interspecific inversion identified between RBT and WCT (Flores et al. 2025; Table 1). This mirrored structural differentiation across homeologous, sex‐linked regions raises the possibility that sex‐biased survival or transmission of RBT_A_ haplotypes could occur in RBT × WCT hybrids. In RBT, segregation distortion at this locus has been observed. In a hatchery setting, RBT_AR_ × RBT_AR_ crosses produced an excess of RBT_AR_ males and RBT_AA_ females (Beulke et al. 2023). Furthermore, the phenotypic expression of variable life histories based on Chr05 complex genotype exhibits sex‐dependent dominance (Pearse et al. 2019; Rundio et al. 2021; Kobayashi et al. 2024). If the transmission and fitness effects of these inversions are indeed intrinsically linked to sex in RBT, it is plausible that the sex of the RBT or WCT parent could disproportionately influence the viability of hybrid offspring. Consequently, this genetic architecture may explain the asymmetric outcomes sometimes observed in wild interspecific crosses, where pairings between male RBT and female WCT appear heavily favoured (Rubidge and Taylor 2004; Kozfkay et al. 2007; McKelvey et al. 2016).

Controlled RBT × WCT crosses involving known Chr05 genotypes are rare (a cross discussed in Flores et al. 2025 represents the only example), and none has accounted for structural variation prior to fertilization. While early work found a general absence of developmental incompatibility in first‐generation crosses (Ferguson et al. 1985), several experiments reported substantial hybrid mortality—including crosses involving female WCT and reciprocal pairings (Leary et al. 1995; Bear 2005; Allendorf et al. 2004)—and none extended beyond the F1 generation. Because F1 hybrids retain complete parental chromosome complements, fitness reductions associated with Chr05 incompatibilities may not manifest until later generations (Edmands 2007; Tymchuk et al. 2007; Ostberg et al. 2011), allowing RBT_A_‐bearing F1s to appear viable despite reduced growth, development, or reproductive success. Resolving these uncertainties will require reciprocal crosses with individuals of known Chr05 genotype extended beyond the F1 generation to quantify sex‐ and genotype‐dependent survivorship, segregation distortion, and the fitness consequences of alternative structural arrangements. Similarly, additional studies that examine the frequency and distribution of Chr05 haplotypes in other RBT × cutthroat trout hybrid zones are needed to evaluate whether these observed patterns are consistent or limited to our study area and current sampling design.

### 4.5 Genomic Insights Into Hybridization and Conservation

Overall, our findings demonstrate how emerging genomic resources can resolve long‐standing questions about hybridization, even in well‐studied evolutionary systems. RBT × WCT hybridization has been studied intensively for decades (Allendorf and Leary 2026), but the integration of dense genomic data with chromosome‐scale reference genomes, structural variation, and local ancestry inference has begun to reveal mechanisms that were difficult to resolve using genome‐wide ancestry estimates alone. More broadly, this study provides a roadmap for disentangling complex hybridization across taxonomic scales, including simultaneous admixture among species and among divergent subspecific lineages within species. Specifically, we found that structural variants, including inversions and Robertsonian rearrangements, appear to act as genomic filters that constrain or facilitate introgression depending on genetic and environmental context. These variants can influence dispersal, suppress recombination, maintain linked ancestry blocks, and potentially contribute to variation in hybrid fitness and survival. By identifying genomic regions that contribute disproportionately to hybrid incompatibilities and ancestry persistence, these approaches can help explain why some hybrid genotypes spread while others are consistently absent despite ongoing gene flow. Similar advances may emerge in other non‐model hybrid systems as chromosome‐scale genomes and increasingly detailed genomic resources become available, revealing mechanisms that remain obscured even in otherwise well‐characterized systems. From a conservation perspective, resolving these mechanisms can improve assessments of hybridization risk by identifying populations, genomic regions, and introduced lineages that disproportionately contribute to the spread of non‐native ancestry or genomic incompatibilities. Genomic approaches therefore provide a powerful framework for linking genome architecture to ecological and evolutionary outcomes and for addressing questions that have challenged managers and researchers for decades.

## Author Contributions

C.D.W. participated in study design, analysed the data, interpreted the data, and wrote the manuscript. S.R.S., M.K., S.J.A., R.P.K., and A.R.W. participated in study design, data generation, data analysis, and data interpretation. B.S., J.D., S.R., and J.N. participated in study design and collected the samples. A.L. and S.P. performed wet lab work and generated the genetic data. P.A.H. provided computational resources and participated in data interpretation. M.C.B. participated in study design and provided funding. All authors contributed to drafting and finalizing the manuscript.

## Funding

This research was supported by funding from the Bonneville Power Administration grant number 199500400 to Montana Fish, Wildlife & Parks.

## Ethics Statement

The present study complies with applicable laws on sampling from natural populations and animal experimentation (ARRIVE guidelines). All received samples were processed following a defined tissue use protocol (TUP 001‐22AWWB) through the University of Montana Institutional Animal Care and Use Committee.

## Conflicts of Interest

The authors declare no conflicts of interest.

## Supporting information


**Table S1:** Quantities of diagnostic SNP loci that distinguish species, subspecies, and orientations of interspecific and intraspecific inversions by the taxon for which they are informative. The chromosome 5 inversion complex had both interspecific and intraspecific diagnostic sets that were independent from one another; rainbow trout (RBT) subspecies loci are a single set of 94 markers that distinguish intraspecific inversion orientation. A similar notion applies to the RBT chromosome 20 (Omy20) intraspecific diagnostic loci. Taxa are coded as follows: RBT = rainbow trout; IRT = interior redband trout; CRT = coastal rainbow trout; WCT = westslope cutthroat trout; YCT = Yellowstone cutthroat trout.
**Table S2:** Linkage map summary.
**Table S3:** (a–d) Ancestry and inversion diagnostic results at the level of collection. Collection metadata, average ancestry proportions for all three global ancestry estimation methods, and counts of inversion interspecific diagnostics within each collection by parental or hybrid genotype are provided. It should be noted that RBT ancestry values are the collapsed proportions attributed to the two RBT subspecies examined (S3b and S3d).
**Table S4:** Diagnosis of chromsome 5 double inversion complex genotypes derived from PCA (Figure 4) for each Kootenai River sample. Group labels correspond to Table S3a–d. Species abbreviations for genotypes are as follows: RBT = Rainbow Trout; WCT = Westlope Cutthroat Trout; YCT = Yellowstone Cutthroat Trout. Haplotypes are coded as follows: A = ancestral orientation; R = rearranged/derived. Recombinant genotypes across inversions are denoted with a semicolon designating a separation of inversion 1 and inversion 2. Recombinants within inversions are denoted as a multiplication symbol.
**Table S5:** Comparison of pairwise *F*_ST_ between taxa across three distinct genomic partitions: the chromosome 5 inversion complex (Chr05 complex), the colinear non‐inverted background of chromosome 5 (Chr05 background), and the rest of the genome (Genome‐wide, excluding chromosome 5). *F*_ST_ was calculated in 500‐kb non‐overlapping windows. Descriptive statistics for *F*_ST_ are presented as Mean ± Standard Deviation (Median). The significance of the difference between the Chr05 complex and both background regions was assessed using a one‐tailed Wilcoxon rank sum test for each taxon pair. The magnitude of these differences is reported as an effect size (Cliff's Delta), followed by the *p*‐value. Dashes (—) indicate baseline background regions against which the Chr05 complex was statistically compared. Taxa are coded as follows: CRT_AA_ = coastal rainbow trout (homozygous ancestral Chr05 genotype); CRT_RR_ = coastal rainbow trout (homozygous rearranged Chr05 genotype); IRT = interior redband trout; WCT = westslope cutthroat trout; YCT = Yellowstone cutthroat trout.
**Figure S1:** A graphical depiction of syntenic chromosomes between (a) Arlee strain rainbow trout (representative of some coastal rainbow trout; CRT) with the homozygous ancestral arrangement (RBT_AA_) of the chromosome 5 inversion complex (Chr05 complex), (b) Arlee strain CRT with the homozygous rearranged form (RBTRR) of the Chr05 complex, (c) interior redband trout (IRT) native to the Kootenai River Basin, (d) westslope cutthroat trout (WCT), and (e) Yellowstone cutthroat trout (YCT). Haploid chromosome counts are listed with each taxon and chromosome numbers are presented in black for RBT taxa, red for WCT, and green for YCT. Chromosomes harbouring interspecific or intraspecific inversions are rotated 90 degrees relative to chromosomes without inversions to illustrate the approximate location of inversions and their synteny with other taxa. Inversions are denoted as a segment of a chromosome bounded by vertical dotted lines. Hatched patterns across the inversion region denote whether a taxon carries the rearranged/derived form of the inversion (R); a lack of hatched patterning indicates the presence of the ancestral form of the inversion (A). Crossed dotted lines connecting two taxa at an inversion denote that they are inverted relative to one another. This figure is adapted from in Flores et al. (2025) with chromosome numbers and the inferred karyotype of YCT being derived from their work. This figure was created using BioRender.
**Figure S2:** A visual representation of the SNPs used in this study and their positions in the Arlee strain Rainbow Trout genome (NCBI: GCF_013265735.2; Gao et al. 2021). Light grey bars onto which tick marks are plotted represent the full physical extent of each chromosome. Sets represented include (a) all 189,984 SNPs and diagnostic SNP sets for (b) rainbow trout (*Oncorhynchus mykiss*; RBT), (c) westslope cutthroat trout (*O. lewisi*; WCT), (d) Yellowstone cutthroat trout (*O. virginalis bouvieri*; YCT), (e) interior redband trout (*O. m. gairdneri*; IRT), and (f) coastal rainbow trout (*O. m.* ssp.; CRT). The set and number of markers are specified on each plot.
**Figure S3:** Manhattan plots of pairwise Weir and Cockerham's *F*_ST_ calculated in non‐overlapping 500‐kb windows using VCFtools with inversion‐aware reference groups, subset to equal sample sizes (N = 31). Comparisons are shown for coastal rainbow trout (CRT) with homozygous ancestral and derived Chr05 complex genotypes (CRT_AA_ and CRT_RR_), interior redband trout (IRT), westslope cutthroat trout (WCT), and Yellowstone cutthroat trout (YCT).
**Figure S4:** Alignment of the global consensus IRT genetic map to the (a) Arlee RBT (NCBI: GCF_013265735.2; Gao et al. 2021), (b) Swanson RBT (NCBI: GCA_025558465.1; Ali et al. 2025), and (c) Connor Lake WCT (NCBI: GCF_045791955.1; Flores et al. 2025) reference genomes. For each IRT linkage group (LG01–LG29), genetic position in the global consensus map (cM; y‐axis) is plotted against physical position on the corresponding reference chromosome (Mb; x‐axis). Each point represents a mapped marker aligned to the reference genome; blue and red points indicate alignments to the positive (+) and negative (−) strands, respectively. Differences in marker order, orientation, and correspondence among linkage groups and reference chromosomes reveal karyotypic differences and candidate structural rearrangements among genomes. Chromosome labels follow the nomenclature of the respective reference genome. Complete chromosome‐by‐chromosome plots for panels a–c are provided as separate supplementary PDF files accompanying this document.
**Figure S5:** Comparisons of ancestry estimates with bar graphs for (a) pairwise Pearson correlation coefficient (*r*) values and (b) pairwise root mean square error (RSME) values comparing each global ancestry method result for each ancestry category examined in this study: Diagnostics = normalized proportions derived from species‐ and subspecies‐diagnostic locus sets; ADMIXTURE = supervised ADMIXTURE v1.3.0 (Alexander et al. 2009); ELAI = global ancestry proportions derived from the program Efficient Local Ancestry Inference (ELAI) v1.01 (Guan 2014). Taxa for which proportions were generated are coded as follows: coastal rainbow trout = CRT; IRT = interior redband trout; westslope cutthroat trout = WCT; Yellowstone cutthroat trout = YCT.
**Figure S6:** Sensitivity of hybrid classification to linkage disequilibrium pruning. Hybrid index (proportion of RBT ancestry using RBT species‐diagnostic loci) plotted against interspecific heterozygosity for RBT × WCT hybrids using an LD‐pruned diagnostic SNP panel. LD pruning retained 103 of 11,071 overlapping RBT‐diagnostic SNPs. Hybrid index and interspecific heterozygosity remained highly correlated with estimates from the unpruned dataset (*r* = 0.9998 and *r* = 0.9895, respectively), and all eight identified F1 individuals retained intermediate hybrid indices and high interspecific heterozygosity. Classification criteria are identical to those used for Figure 3.
**Figure S7:** Chromosome painting plots showing the population‐level consensus of ancestry blocks—excluding homozygous inland redband trout (IRT)—estimated by the program Efficient Local Ancestry Inference (ELAI) (Guan 2014) across all individuals in two collections from lower Bear Creek in the Libby Creek Drainage: (a) Bear_20 and (b) Bear_21. Ancestry blocks are mapped to the Arlee strain rainbow trout reference genome (NCBI: GCF_013265735.2; Gao et al. 2021) and the total length of each chromosome is represented by an underlying black dotted line. Homozygous coastal rainbow trout (CRT) and westslope cutthroat trout (WCT) ancestry blocks are found in some portions of the genome, but hybrid ancestry primarily occurred as heterozygous blocks. Interspecific inversions are depicted as hollow black boxes and include the Chromosome 5 (Omy05) complex which displays intraspecific polymorphism between CRT and IRT. Despite low global CRT ancestry proportions (3.80% in Bear_20 and 4.66% in Bear_21), much of the genome is affected by CRT hybridization across both collections.
**Figure S8:** Scatterplots showing the relationship between inversion‐specific heterozygosity (x‐axis) and chromosome‐wide heterozygosity excluding the inversion (y‐axis) at rainbow trout (RBT) diagnostic loci. Pearson correlation coefficients (*r*) are shown. Points are coloured by inferred hybrid class with hybrids beyond the F1 (RBT × WCT) hybrid class noted as a backcross with majority RBT, westslope cutthroat trout (WCT), or Yellowstone cutthroat trout (YCT) ancestry. Relative to RBT, WCT have a fusion of RBT chromosomes 20 (Omy20) and 28 (Omy28) which forms WCT chromosome Ocl20 (20/28 fusion). This fusion also exists in YCT (Flores et al. 2025; Ostberg et al. 2013). These plots show evidence of recombination suppression across Omy28 when the Chr20 interspecific inversion is present in hybrid individuals that are Robertsonian heterozygotes. The Chr20 inversion was already shown to suppress recombination across Omy20 in Figure 3 (Pearson's *r* = 0.96), indicating that this inversion and Robertsonian heterozygosity may both contribute to strong recombination suppression across both RBT chromosomes in interspecific hybrids. In contrast, Chr20 inversion heterozygosity was not associated with recombination suppression on chromosome 10 (Chr10), which has no associated fusion. Evidence of free recombination on Chr10 was observed regardless of Chr20 inversion presence.
**Figure S9:** Scatterplots showing the relationship between heterozygosity across rainbow trout (RBT) chromosome Arm 1 (x‐axis) and RBT chromosome Arm 2 (y‐axis) at RBT‐diagnostic loci for those RBT chromosomes that correspond to fissions in westslope cutthroat trout (WCT) as seen in Figure S1. Pearson correlation coefficients (*r*) for each structural variant are shown. Points are coloured by inferred hybrid class with hybrids beyond the F1 (RBT × WCT) hybrid class noted as a backcross with majority RBT, westslope cutthroat trout (WCT), or Yellowstone cutthroat trout (YCT) ancestry. Recombinants were observed at a lower frequency for these RBT chromosomes than for chromosome 1 (Chr01), which is not associated with rearrangements in cutthroat trout relative to RBT. Heterozygosity across Arm 1 was strongly associated with heterozygosity across Arm 2 for all WCT fissions, indicating that Robertsonian heterozygotes in hybrids are likely resulting in recombination suppression but it is weaker than suppression from interspecific inversions.
**Figure S10:** Chromosome painting plots showing the population‐level consensus of ancestry blocks—excluding homozygous interior redband trout (IRT)—estimated by the program ELAI (Guan 2014) across all individuals in two IRT populations believed to be naturally introgressed with westslope cutthroat trout (WCT) and isolated from non‐native taxa: (a) upper East Fork Yaak River (EF_Yaak_20, EF_Yaak_21, EF_Yaak_22, and EF_Yaak_24) and (b) upper Bear Creek (Bear_17, Up_Bear_22, and Bear_24). Blocks are mapped to the Arlee strain rainbow trout reference genome (NCBI: GCF_013265735.2; Gao et al. 2021). Interspecific inversions are depicted as hollow black boxes. The EF Yaak consensus displays admixture that affects the majority of the genome and occurs in large, contiguous blocks driven by recent, ongoing hybridization. In contrast, the Upper Bear consensus exhibits a highly fragmented pattern typical of historical introgression, where trace IRT × WCT ancestry (IRT/WCT heterozygous blocks) is scattered across colinear regions but is completely absent within interspecific inversions.
**Figure S11:** Relationship between the number of interspecific heterokaryotypic inversions and genome‐wide admixture proportions in Kootenai Basin Oncorhynchus hybrids. Minor parent ancestry (x‐axis)—calculated as the minimum of the rainbow trout (RBT) or westslope cutthroat trout (WCT) ELAI ancestry proportions—serves as a proxy for the extent of backcrossing. The y‐axis displays the per‐individual count of heterokaryotypic inversions (0–6), explicitly separating the Chr05 double inversion complex into two independent variants alongside Chr17, Chr20, Chr22, and Chr29. (a) Jittered scatterplot of the continuous relationship between minor parent ancestry and inversion count, with a LOESS regression line (grey shading indicates 95% confidence interval). (b) Boxplots displaying the distribution of heterokaryotypic inversions binned by admixture categories representing extent of backcrossing, overlaid with raw data points. Both panels illustrate the loss of structural heterozygosity during repeated backcrossing.

## Data Availability

Data Accessibility: Raw sequence data (sample fastqs) for linkage mapping and landscape‐level analyses can be found under BioProject accession PRJNA1510277. The filtered variant call format file and associated sample metadata used in population‐level analyses are available via Zenodo: https://doi.org/10.5281/zenodo.21726414. Scripts and supporting files necessary to reproduce the analyses are also available via Zenodo and GitHub: https://github.com/cdwgen/RBT_WCT_Inversion_Polymorphisms. Benefits from this research accrue from the sharing of our data and results on public databases as described above.
