# Supplementary material for: Inversion Polymorphisms Carried by Introduced Rainbow Trout Disrupt Hybrid Zones Between Native Species
Source: Mol Ecol. 2026 Sep 20;35(18):e70556. doi: 10.1111/mec.70556 (PMC13591004; doi:10.1111/mec.70556)
Supplement: Supplementary file 1 [file MEC-35-e70556-s001.docx]

**Supplementary Information for:**

**Inversion Polymorphisms Carried by Introduced Rainbow Trout Disrupt Hybrid Zones Between Native Species**

Craig D. Wells, Seth R. Smith, Marty Kardos, Stephen J. Amish, Angela Lodmell, Sally Painter, Brian Stephens, James Dunnigan, Scott Relyea, Jason Nachtmann, Paul A. Hohenlohe, Matthew C. Boyer, Ryan P. Kovach, Andrew R. Whiteley

**Correspondence:** [craigdwells622@gmail.com](mailto:craigdwells622@gmail.com)

| **TABLE OF CONTENTS** | |
| --- | --- |
| **SUPPLEMENTARY METHODS** |  |
| Genetic Map Construction | Page 3 |
| Notes on Dataset Filtering | Page 4 |
| Identification of Reference Groups for Diagnostic Locus Discovery | Page 4 |
| Species- and Subspecies-Diagnostic Loci | Page 4 |
| Chromosomal Inversion-Diagnostic Loci | Page 5 |
| Hybrid Class Inference | Page 6 |
| Sensitivity to Linkage Disequilibrium Pruning | Page 6 |
| **SUPPLEMENTARY RESULTS** |  |
| Examination of Interspecific and Intraspecific Inversions | Page 6 |
| Comparison of Global Ancestry Estimation Methods | Page 7 |
| Sensitivity to Linkage Disequilibrium Pruning | Page 7 |
| Admixture in Hatchery Strains | Page 8 |
| Notes on the Chr20 Intraspecific Inversion | Page 8 |
| **SUPPLEMENTARY TABLES** |  |
| Table S1 | Page 12 |
| Table S2 | See additional materials |
| Table S3 | See additional materials |
| Table S4 | See additional materials |
| Table S5 | Page 14 |
| **SUPPLEMENTARY FIGURES** |  |
| Figure S1 | Page 15 |
| Figure S2 | Page 16 |
| Figure S3 | Page 23 |
| Figure S4 | See additional materials |
| Figure S5 | Page 25 |
| Figure S6 | Page 26 |
| Figure S7 | Page 28 |
| Figure S8 | Page 29 |
| Figure S9 | Page 30 |
| Figure S10 | Page 32 |
| Figure S11 | Page 33 |

SUPPLEMENTARY METHODS

Genetic Map Construction

A consensus linkage map for interior redband trout (IRT) was constructed using 4 gynogenetic haploid and 6 diploid families. Population-specific maps for three Kootenai Basin populations were also constructed using subsets of these data. Haploid family mothers were sourced from the East Fork Yaak River (EF Yaak) and Wolf Creek drainages (Figure 1). Diploid families consisted of one family with two EF Yaak parents, one family with two Wolf Creek parents, one family with a Wolf Creek mother and an EF Yaak father, one family with an EF Yaak mother and a Wolf Creek father, and two families with two Gerrard strain (discussed below) parents sourced from Murray Springs Hatchery. Diploid families each consisted of 91 offspring, while haploid families included from 16 to 108 offspring.

Haploid families were created by fertilizing eggs with UV-irradiated milt, which renders sperm genetically inactive so that no paternal chromosomes contribute to the developing embryo (Chourrout 1982). Fertilized eggs were incubated until the eyed stage and preserved in 95% ethanol prior to genomic analysis. Genotype data were generated using an existing RAD-capture panel as described below, except that RAD haplotype genotypes exported by STACKS v2.58 (Catchen et al. 2013) were used for linkage mapping rather than SNP genotypes.

To verify ploidy, we plotted the frequency of non-maternal alleles against multi-locus heterozygosity for all individuals and excluded any putatively haploid offspring whose values resembled those of diploid families. For haploid families, loci were classified as singletons or confounded duplicates, and genotypes were called for confounded duplicates with mappable segregation following Waples et al. (2016). For diploid families, we excluded loci identified as confounded duplicates, loci with more than 4 alleles in any single family, loci where neither parent was heterozygous, and loci with missing parental genotypes. Offspring genotypes incompatible with parental genotypes were tallied by locus, loci with more than 2 impossible genotypes were discarded, and remaining impossible genotypes were treated as missing data. Chi-square tests were then used to assess each locus for departures from Mendelian expectations, and loci with significant deviations were discarded following false discovery rate correction (Benjamini & Hochberg 1995; *q* < 0.1).

We used LepMap3 (Rastas 2017) to construct the consensus IRT linkage map. *SeparateChromosomes2* was run across LOD scores ranging from 3 to 40 while restricting the analysis to markers that were informative in male parents. All LOD thresholds between 3 and 20 recovered the expected number of linkage groups (1n = 29), so a threshold of LOD = 3 was adopted for marker clustering. Female informative, double informative, and unassigned male informative markers were then incorporated into the framework map using *JoinSingles2All*, starting with a LOD threshold of 60 and a LOD difference threshold of 3. The LOD threshold was reduced by 3 at each iteration until we reached a final value of 3.

Marker ordering was performed using *OrderMarkers2* with *minError* set to 0.001, the recombination parameter set to 10⁻⁶, and *numMergeIterations* set to 1. Twenty independent iterations were run per linkage group and the order with the highest likelihood was retained. A second run of OrderMarkers2 was then carried out using the maximum likelihood order as input, with *numPolishIterations* set to 3 and *numMergeIterations* set to 6 to further refine marker positions. Population-specific maps were constructed by masking markers informative only in parents from non-target populations and repeating the map-building process described above.

Identification of Reference Groups for Diagnostic Locus Discovery

As stated in the main text, identification of reference groups for downstream analyses followed similar methodology to Matteucci et al. (2019). Patterns of genetic differentiation among samples were explored using genotype-based Principal Component Analysis (PCA) in R (R Core Team 2019). Missing data were imputed to locus-specific means and a PCA (centered, unscaled) was generated using the package adegenet v2.1.11 (Jombart 2008; Jombart and Ahmed 2011). Clusters identified in the PCA, along with sample provenance, were used to assign samples to putative rainbow trout (RBT), westslope cutthroat trout (WCT), and Yellowstone cutthroat trout (YCT) species reference groups. PCAs of pairwise combinations of the three species clusters were used to refine sample groups to individuals least likely to be hybrids based on position in PCA space. Input files were created using the *--make-bed* option of PLINK v2.0 (Chang et al. 2015) and PCA-derived sample lists were used to run 10 replicates with randomized seeds of unsupervised ADMIXTURE v1.3.0 (Alexander et al. 2009) for each pairwise species grouping (*K* = 2) to further refine reference group assignment. Samples (excluding any technical replicates) with *Q*-values ≥0.999 for a single species group were retained for diagnostic locus discovery.

This process was similarly repeated to identify subspecies reference groups within the RBT species reference samples. Clusters identified in a PCA of all samples identified as *Q* ≥ 0.999 RBT, alongside sample provenance, were used to categorize putative coastal rainbow trout (CRT) and IRT samples. Again, 10 replicates with randomized seeds of unsupervised ADMIXTURE (*K* = 2) were used to refine reference group assignments. Samples (excluding any technical replicates) where *Q* ≥ 0.999 for a single subspecies group were retained for genome-wide diagnostic locus discovery.

Species- and Subspecies-Diagnostic Loci

To identify species-diagnostic loci, we estimated genome-wide allele frequencies for reference groups of RBT, WCT, and YCT using the *--freq* option in VCFtools. Because reference sample sizes were uneven among taxa, we standardized sampling effort by randomly subsampling the larger groups (RBT and WCT) to match the smallest (YCT; *N* = 51). This was done to ensure the stringency of the diagnostic filter—and the baseline probability of falsely retaining a shared ancestral polymorphism—was symmetrical across all three lineages. This procedure was repeated 30 times. Replicate allele frequency estimates were highly consistent (mean *SD* across loci = 0.0065 for RBT and 0.0027 for WCT), so replicate datasets were merged by averaging allele frequencies across replicates. We then identified diagnostic loci based on absolute allele frequency differences between taxa, testing thresholds of 0.95, 0.975, and 0.99. Lower thresholds increased the number of loci classified as diagnostic but also increased isolated singletons that were randomly distributed across the genome and did not form contiguous ancestry tracts, consistent with inclusion of ancestral polymorphisms (Racimo et al. 2015). The most stringent threshold (0.99) reduced detection of small introgressed blocks. Because broad-scale ancestry patterns were stable across thresholds, and given that low marker density makes distinguishing short introgressed tracts from isolated ancestral polymorphisms inherently difficult, we selected the intermediate cutoff (≥0.975) as a conservative balance between minimizing false positives from ancestral polymorphism and retaining power to detect small hybrid ancestry tracts.

This approach was replicated to identify subspecies-diagnostic SNPs for IRT and CRT. Groups of IRT, CRT, WCT, and YCT reference individuals (*N* = 51) were randomly subsampled and allele frequencies were estimated for each of 30 replicates. Again, allele frequency estimates were highly consistent across replicates (mean *SD* across loci = 0 for CRT, 0.0041 for IRT, 0.0065 for RBT, and 0.0027 for WCT) so all replicates were averaged together. We then identified diagnostic loci for IRT and CRT based on absolute allele frequency differences between either subspecies and all other groups. Again, we tested thresholds of 0.95, 0.975, and 0.99. Loci defined at the freq ≥ 0.99 threshold resolved differentiation between IRT and CRT groups best and this set was used in all subsequent analyses. High similarity of IRT and CRT across the majority of the genome (Figure S3) meant that more relaxed frequency thresholds primarily added loci that were scattered across the genome and did not cluster into contiguous blocks, a pattern consistent with ancestral polymorphisms. Given the low divergence between IRT and CRT, limited marker density further reduces the ability to distinguish these isolated loci from short introgressed tracts, supporting use of the more stringent ≥0.99 threshold for downstream analyses.

Chromosomal Inversion-Diagnostic Loci

Putative interspecific inversions identified during genome structure analysis were examined using PCA. A VCF file of all samples was subset to genomic coordinates for each putative inversion (Table 1) using position filtering options in VCFTools followed by creation of PCA plots for each subset. We expected individuals to segregate along PC1 into two homokaryotypic species groups with heterokaryotypic individuals in between, as in Knief et al. (2024). Random regions of the genome of similar length with no known structural variation were examined for similar patterns but these showed more proximate RBT and Cutthroat Trout clusters with non-distinct intermediates and typically had lower variation displayed along PC1. Diagnostic locus discovery for interspecific inversions involved retrieving loci completely fixed for only one taxon (freq = 1.0) between the RBT, WCT, and YCT reference groups using previously generated frequency files.

Intraspecific inversions were explored and diagnosed in a similar way. A VCF of all previously identified RBT species reference samples was subset to genomic coordinates for each putative inversion (Pearse et al. 2019; Hale et al. 2024; Ali et al. 2025) using position filtering options in VCFTools and PCA plots were created. Segregation of homozygous and heterozygous genotypes followed the same pattern as in interspecific comparisons. Sample coordinates from the PCAs were then used to create lists of homozygous reference groups. Allele frequencies for all loci across the inversion regions were calculated for each reference group using *vcftools --freq* and completely fixed loci (freq = 1.0) between homozygous forms were retained as diagnostic loci.

Genotypes were extracted for each set of inversion-specific diagnostic loci from the filtered VCF of all samples, recoded by allele state, and summarized to quantify the proportion of alleles matching the taxon or homozygous inversion genotype for each inversion. These results were normalized and used to diagnose the genotype of each individual. For interspecific inversions, heterokaryotypic individuals were denoted as having a perfect or near-perfect split of diagnostic locus proportions (~0.5) between taxa across the region of interest. Proportions outside this split or further divisions between more than two taxa were taken as evidence of recombination between some taxa and the possibility that recombining taxa share the same form of the inversion. For intraspecific inversions, heterozygous individuals were denoted by having proportions of diagnostic loci split cleanly between the two homozygous forms of the inversion.

Hybrid Class Inference

To further resolve hybrid classes strictly between RBT and WCT (excluding individuals with >0.1% YCT ancestry), the proportion of RBT ancestry (pRBT) at diagnostic loci (hybrid index) was plotted against interspecific heterozygosity (*H*_I_; calculated as the proportion of heterozygous genotypes across all valid diagnostic loci). RBT-diagnostic loci with a frequency >0.99 were used for this analysis to maintain consistency with recombination suppression analyses. Individuals were assigned to the following classes: unadmixed WCT (pRBT ≤ 0.01), unadmixed RBT (pRBT ≥ 0.99), F1 hybrids (confirmed via structural variants and bounded by 0.40 ≤ pRBT ≤ 0.60 and *H*_I_ > 0.80), and F2 hybrids (0.40 ≤ pRBT ≤ 0.60 and 0.40 ≤ *H*_I_ ≤ 0.60). To distinguish strict directional backcrosses from later-generation hybrids, we adapted the geometric boundaries described by Rosenthal et al. (2022), anchoring backcrosses to a ±0.1 tolerance band along their theoretical maximum heterozygosity limits. Specifically, backcrosses to WCT were bounded by *H*_I_ – 2(pRBT) = ±0.1, and backcrosses to RBT were bounded by *H*_I_ - 2(1-pRBT) = ±0.1. All remaining admixed individuals falling outside these boundaries were categorized as complex admixture.

Sensitivity to Linkage Disequilibrium Pruning

To evaluate whether retention of linked loci affected hybrid classification or global ancestry estimates, we repeated the hybrid triangle plot and supervised ADMIXTURE analyses using a dataset pruned for linkage disequilibrium (LD). The genome-wide SNP dataset was pruned in PLINK v2.0 using a 50-SNP window, a 10-SNP step, and an *r*^2^ threshold of 0.1 (*--indep-pairwise 50 10 0.1*), as recommended for ADMIXTURE (Alexander et al. 2009). For the triangle plot, retained SNP positions were intersected with the RBT species-diagnostic loci and hybrid index, interspecific heterozygosity, and hybrid class were recalculated using the same criteria as the primary analysis. Supervised ADMIXTURE was repeated using the LD-pruned dataset with the same reference individuals and *K* = 4.

SUPPLEMENTARY RESULTS

Examination of Interspecific and Intraspecific Inversions

As downstream RAD-Capture analyses were conducted with alignment to the Arlee assembly, an alignment of Arlee to the WCT assembly was performed. In addition to the known chromosome 20 (Chr20), chromosome 22 (Chr22), and chromosome 29 (Chr29) interspecific inversions, two smaller inversions on chromosomes 13 (Chr13) and 17 (Chr17) not described in Flores et al. (2025) were recorded (Table 1). It should be noted that this apparent Chr17 inversion matches the coordinates of the intraspecific Chr17 inversion described in Ali et al. 2026. Arlee and WCT were also aligned to the Swanson assembly to confirm that chromosome 5 double inversion complex (Chr05 complex) coordinates were consistent between IRT, CRT, and WCT (Table 1).

Principal component analyses for each inversion region displayed the 3 band pattern expected for chromosomal inversions except for the Chr13 inversion, which was highly variable among species and within populations, rendering it uninformative for species or subspecies delimitation (<https://cdwgen.github.io/redband-trout-hybridization/Interspecific_Inversions/>). For all other inversions, we identified hundreds of taxon-specific diagnostic loci (Table S1). Tests of inversion polarity revealed that RBT carry the ancestral form of the Chr17 and Chr29 inversions. No RBT populations examined in this study showed evidence of intraspecific variability at the Chr17 inversion, thus we treat it as an interspecific inversion though results of Ali et al. 2026 suggest that our dataset may lack the geographic representation necessary to detect intraspecific variation and linkage mapping analyses indicate this region may have greater complexity. WCT carry the ancestral form of the Chr20 and Chr22 inversions and this form is likely shared by YCT based on apparent recombination between Cutthroat taxa at diagnostic loci (Table 1), placement in PCA space, and previous observations by Ostberg et al. (2013). The orientations of the Chr20 and Chr22 inversions in the RBT taxa relative to Atlantic Salmon match previous observations about RBT in Sutherland et al. (2016). The Omy20 intraspecific inversion was detectible but overlap with the Chr20 interspecific inversion and apparent rarity in the study system complicated inferences (see below).

Comparison of Global Ancestry Estimation Methods

Pairwise comparisons evaluating relative concordance among all three global ancestry estimation methods indicated very high agreement (Pearson’s *r* > 0.99) across all estimates (Figure S5a). Root mean square error (RMSE) between each pair of methods was low for RBT, WCT, and YCT (< 0.005) and slightly higher for CRT and IRT (0.028–0.049) (Figure S5b). The higher variability in RBT subspecies estimates is consistent with the reduced resolution of frequency-based clustering approaches when allele frequency differentiation among groups is low (Raj et al. 2014). Furthermore, frequency-based models such as ADMIXTURE can exhibit bias in complex admixture scenarios relative to haplotype-based approaches, which leverage linkage disequilibrium to improve ancestry estimation accuracy (Uren et al. 2020).

To evaluate model accuracy, we treated our diagnostic loci estimates as the empirical baseline. When compared to this baseline, ADMIXTURE exhibited slightly lower error for the major ancestry components (RBT, WCT, and YCT; RMSE = 0.001–0.003) than ELAI (RMSE = 0.002–0.005). This could be due to ADMIXTURE missing trace admixture, effectively smoothing these estimates toward 1.0 or 0.0, which artificially minimizes variance across large, non-hybridized genomic regions. ELAI showed stronger concordance with diagnostic loci in the detection of small hybrid ancestry blocks and reduced error for the minor CRT and IRT components (*r* = 0.995–0.998, RMSE = 0.028) relative to ADMIXTURE (RMSE = 0.048–0.049), though some erroneous classification of ancestry blocks is expected due to linkage disequilibrium decay, limited differentiation among lineages, and finite marker density. Given that ELAI aligned closely with diagnostic estimates while leveraging information from a larger number of loci to provide higher resolution—particularly for IRT and CRT lineages where diagnostic markers are limited—we deemed ELAI results to be the most reliable estimate of global ancestry.

Sensitivity to Linkage Disequilibrium Pruning

After LD pruning and subsetting to overlapping RBT species-diagnostic SNPs, 103 of 11,071 loci were retained for repeating the triangle plot analyses. Estimates from the unpruned and pruned analyses were highly correlated (*r* = 0.9998 for hybrid index and *r* = 0.9895 for interspecific heterozygosity). Hybrid class assignments were unchanged for 2,264 of 2,473 Kootenai Basin individuals (91.55%). Of the 209 individuals for which a change was observed, 200 involved movement between an unadmixed category and the adjacent backcross category, reflecting small shifts across the strict classification thresholds as unadmixed (≤0.01 or ≥0.99). All eight identified F1 individuals retained intermediate hybrid indices and high interspecific heterozygosity. Thus, LD pruning reduced marker resolution without changing the overall triangle pattern or its interpretation (Figure S6).

The full LD-pruned dataset retained 52,424 SNP positions (27.6% of all SNPs). Supervised ADMIXTURE using this dataset yielded correlations between the unpruned and pruned ancestry estimates of 0.9993 to 1.0000 across the four ancestry components. The ancestry component with the highest inferred contribution was unchanged for 99.7% of individuals, and 98.8% of individuals differed by less than 0.05 for every component. For a known RBT×WCT F1 sequencing control that is heterokaryotypic at all inversions, Griz_20_15 (see VCF metadata at <https://doi.org/10.5281/zenodo.21726414>), the unpruned analysis estimated 47.0% RBT and 52.8% WCT ancestry, whereas the pruned analysis estimated 28.1% RBT and 71.8% WCT ancestry. The latter result was reproduced across three independent seeds. Overall, LD pruning produced nearly identical ancestry estimates for most individuals and did not alter the biological conclusions of either the triangle plot or ADMIXTURE analysis.

Admixture in Hatchery Strains

We genotyped samples from two hatchery lines believed to represent non-hybridized upper Columbia River IRT: Pennask Lake Rainbow Trout from Pennask Lake, British Columbia and Gerrard strain Rainbow Trout, whose ancestors originate from the Lardeau River—a tributary of Kootenay Lake, in British Columbia. Though all individuals from these collections had inversion arrangements consistent with Kootenai IRT, CRT admixture was detected in both hatchery lines with all three global ancestry estimates. Local ancestry analyses from ELAI inferred large admixture blocks in many individuals despite low proportions of admixture (2.00% and 1.78% average CRT ancestry in each line).

Notes on the Chr20 Intraspecific Inversion

A secondary RBT intraspecific inversion on RBT chromosome 20 (Omy20 inversion; Hale et al. 2024) is nested within the Chr20 interspecific inversion (Flores et al. 2025), corresponding to positions 5,600,000-19,200,000 bp on Omy20 in the Arlee RBT reference genome (NCBI: GCF_013265735.2; Gao et al. 2021). This inversion is primarily polymorphic in the southernmost distribution of RBT in North America, though it is almost certainly fixed for the rearranged form in IRT populations based on observations in Hale et al. (2024), our linkage maps, and RAD-Capture data. Variability in CRT populations and hybrids with CRT ancestry was anticipated based on Hale et al. (2024). Intraspecific inversion signal in PCA space was weak for this polymorphism and was mainly detectible in interspecific comparisons. Still, 22 diagnostic loci distinguishing the ancestral and rearranged forms in RBT were discovered (Table S1). We observed inversion variability in Eagle Lake hatchery RBT, with this population having the highest frequency of individuals with the homozygous ancestral genotype (Omy20_AA_)—in line with observations of Hale et al. (2024). Few wild fish or other hatchery strains had much variability in their Omy20 genotypes. A total of 29 heterozygotes were observed on the landscape. Only four Omy20_A_ homozygotes were observed across three collections. The greatest occurrence of Omy20_A_ haplotypes was in Howard Lake in the Libby Creek drainage, though all of these fish were heterozygotes (*N* = 9). Stocking records indicate that only Arlee, Kamloops, and FWP “Red Band” strain fish were ever stocked here (Montana Fish, Wildlife & Parks Stocking Records); hatchery lines where this form is not expected. The Omy20_A_ form is colinear to part of Chr20 in the Cutthroat taxa (Ocl20 in WCT) observed in this study, similar to the RBT_A_ form of the Chr05 complex. Because of this, it might be expected to have similar incompatibilities in RBT and Westslope Cutthroat Trout (WCT) hybrids. Only three individuals that were hybrids between Omy20_A_ and Ocl20 individuals were observed. No partial recombinants were observed. Due to the Omy20_R_ inversion being indistinguishable from the RBT form of the Chr20 interspecific inversion, it is hard to know how Omy20_R_ and Ocl20 hybrids would compare in the absence of the Chr20 interspecific inversion.

It could be that the low frequency of Omy20_A_/Ocl20 hybrids is due to some sort of incompatibility, but the low frequency and somewhat non-random distribution of individuals with an Omy20_A_ haplotype in the system suggests that they may have been infrequently planted in the Kootenai Basin. The long-term planting of Arlee strain CRT (which are nearly fixed for the Omy20_RR_ genotype; one individual out of 77, or 1.29%, was detected with a heterozygous Omy20 inversion) and the detection of no Omy20_A_ haplotypes in a sample of fish from Middle Cody Lake that likely descend from a 1934 stocking event (see main text) points to this possibility. Montana Fish, Wildlife & Parks (FWP) did not begin using Eagle Lake strain RBT until after they were brought to the state in 1980 (Hensler 1986). This followed the statewide ban on stocking trout streams with hatchery fish in 1974 (Zackheim 2005), meaning ancestral Omy20 haplotypes could only have been introduced from Eagle Lake stock via planted lakes after this point. However, no Eagle Lake stocking records coincide with the occurrence of this haplotype (Montana Fish, Wildlife & Parks Stocking Records) suggesting that other strains used less frequently than Arlee may have had greater Omy20 genotype variability and this persists in parts of the system. In contrast, the high frequency of RBT_AA_ Chr05 genotypes in the Middle Cody Lake sample and Arlee strain RBT likely means this genotype was widely stocked on the landscape for decades, making the observed habitat stratification in RBT_AA_ individuals and the near-absence of WCT_A_/RBT_A_ hybrids more striking.

REFERENCES

Alexander, D. H., Novembre, J., & Lange, K. (2009). Fast model-based estimation of ancestry in unrelated individuals. *Genome Research*, 19(9), 1655–1664. <https://doi.org/10.1101/gr.094052.109>

Ali, A., Gao, G., Al-Tobasei, R., Youngblood, R. C., Waldbieser, G. C., Scheffler, B. E., Palti, Y., & Salem, M. (2025). Chromosome-level genome assembly and annotation of the Swanson rainbow trout homozygous line. *Scientific Data*, 12(345). <https://doi.org/10.1038/s41597-025-04693-7>

Ali, A., Waldbieser, G. C., Youngblood, R. C., Wheeler, P. A., Scheffler, B. E., Willis, S., Narum, S. R., Thorgaard, G. H., Salem, M., & Palti, Y. (2026). De novo genome assemblies of four rainbow trout genetic lines reveal structural variants in pursuit of a pangenome reference. *Molecular Ecology Resources*, *26*(5), e70167. <https://doi.org/10.1111/1755-0998.70167>

Benjamini, Y., & Hochberg, Y. (1995). Controlling the false discovery rate: a practical and powerful approach to multiple testing. Journal of the Royal Statistical Society: Series B (Methodological), 57(1), 289–300. <https://doi.org/10.1111/j.2517-6161.1995.tb02031.x>

Catchen, J., Hohenlohe, P. A., Bassham, S., Amores, A., & Cresko, W. A. (2013). Stacks: An analysis tool set for population genomics. *Molecular Ecology*, *22*, 3124–3140. <https://doi.org/10.1111/mec.12354>

Chang, C. C., Chow, C. C., Tellier, L. C. A., Vattikuti, S., Purcell, S. M., & Lee, J. J. (2015). Second-generation PLINK: rising to the challenge of larger and richer datasets. *GigaScience*, 4(1). <https://doi.org/10.1186/s13742-015-0047-8>

Chourrout, D. (1982). Gynogenesis caused by ultraviolet irradiation of salmonid sperm. *Journal of Experimental Zoology*, *223*, 175–181. [https://doi.org/10.1002/jez.1402230209](https://www.google.com/search?q=https://doi.org/10.1002/jez.1402230209)

Flores, A.-M., Christensen, K. A., Godin, T., Palti, Y., Campbell, M. R., Waldbieser, G. C., Simpson, S. A., Scheffler, B. E., Smith, S. R., Whiteley A. R., Kovach, R. P., Luikart, G., Boyer, M. C., Kardos, M., Relyea, S., Wells, C. D., & Koop, B. F. (2025). The genome assembly of the westslope cutthroat trout, *Oncorhynchus lewisi*, reveals interspecific chromosomal rearrangements with the rainbow trout, *Oncorhynchus mykiss*. G3: Genes, Genomes, Genetics, 15(5). <https://doi.org/10.1093/g3journal/jkaf064>

Gao, G., Magadan, S., Waldbieser, G. C., Youngblood, R. C., Wheeler, P. A., Scheffler, B. E., Thorgaard, G. H., & Palti, Y. (2021). A long reads-based de-novo assembly of the genome of the Arlee homozygous line reveals chromosomal rearrangements in rainbow trout. *G3: Genes, Genomes, Genetics*, 11:jkab052. <https://doi.org/10.1093/g3journal/jkab052>

Hale, M. C., Pearse, D. E., & Campbell, M. A. (2024). Characterization and distribution of a 14-Mb chromosomal inversion in native populations of rainbow trout (*Oncorhynchus mykiss*). *G3: Genes, Genomes, Genetics*, 14(7), jkae100. <https://doi.org/10.1093/g3journal/jkae100>

Hensler, M. E. (1986). *Food habits, growth rates, and condition factors of three strains of trout*. [Master's thesis, Montana State University]. Bozeman, MT.

Jombart, T. (2008). adegenet: A R package for the multivariate analysis of genetic markers. *Bioinformatics*, 24, 1403–1405. <https://doi.org/10.1093/bioinformatics/btn129>

Jombart, T., & Ahmed, I. (2011). adegenet 1.3-1: New tools for the analysis of genome-wide SNP data. *Bioinformatics*, 27(21), 3070–3071. <https://doi.org/10.1093/bioinformatics/btr521>

Knief, U., Müller, I. A., Stryjewski, K. F., Metzler, D., Sorenson, M. D., & Wolf, J. B. W. (2024). Evolution of chromosomal inversions across an avian radiation. *Molecular Biology and Evolution*, 41(6). <https://doi.org/10.1093/molbev/msae092>

Mattucci, F., Galaverni, M., Lyons, L. A., Alves, P. C., Randi, E., Velli, E., Pagani, L., & Caniglia, R. (2019). Genomic approaches to identify hybrids and estimate admixture times in European wildcat populations. *Scientific Reports*, 9(11612). <https://doi.org/10.1038/s41598-019-48002-w>

Montana Fish, Wildlife & Parks Stocking Records online database. Retrieved 2026. <https://myfwp.mt.gov/fishMT/plants/plantreport>

Ostberg, C. O., Hauser, L., Pritchard, V. L., Garza, J. C., & Naish, K. A. (2013). Chromosome rearrangements, recombination suppression, and limited segregation distortion in hybrids between Yellowstone cutthroat trout (*Oncorhynchus clarkii bouvieri*) and rainbow trout (*O. mykiss*). *BMC Genomics*, 14(570). <https://doi.org/10.1186/1471-2164-14-570>

Pearse, D. E., Barson, N. J., Nome, T., Gao, G., Campbell, M. A., Abadía-Cardoso, A., Anderson, E. C., Rundio, D. E., Williams, T. H., Naish, K. A., Moen, T., Liu, S., Kent, M., Moser, M., Minkley, D. R., Rondeau, E. B., Brieuc, M. S. O., Sandve, M., SR, MR, L., & S. (2019). Sex-dependent dominance maintains migration supergene in rainbow trout. *Nature Ecology & Evolution*, 3, 1731–1746. <https://doi.org/10.1038/s41559-019-1044-6>

R Core Team. (2019). *R: A language and environment for statistical computing*. R Foundation for Statistical Computing. <https://www.r-project.org/>

Racimo, F., Sankararaman, S., Nielsen, R., & Huerta-Sánchez, E. (2015). Evidence for archaic adaptive introgression in humans. *Nature Reviews Genetics*, 16(6), 359–371. <https://doi.org/10.1038/nrg3936>

Raj, A., Stephens, M., & Pritchard, J. K. (2014). fastSTRUCTURE: Variational Inference of Population Structure in Large SNP Data Sets. *Genetics*, *197*(2), 573–589. <https://doi.org/10.1534/genetics.114.164350>

Rastas, P. (2017). Lep-MAP3: robust linkage mapping even for low-coverage whole genome sequencing data. *Bioinformatics*, *33*(23), 3726–3732. <https://doi.org/10.1093/bioinformatics/btx494>

Rosenthal, W. C., Fennell, J. M., Mandeville, E. G., Burckhardt, J. C., Walters, A. W., & Wagner, C. E. (2022). Hybridization decreases native cutthroat trout reproductive fitness. *Molecular Ecology, 31*, 4224–4241. <https://doi.org/10.1111/mec.16578>

Sutherland, B. J. G., Gosselin, T., Normandeau, E., Lamothe, M., Isabel, N., Audet, C., & Bernatchez, L. (2016). Salmonid chromosome evolution as revealed by a novel method for comparing RADseq linkage maps. *Genome Biology and Evolution*, 8, 3600–3617. <https://doi.org/10.1093/gbe/evw262>

Waples, R. K., Seeb, L. W., & Seeb, J. E. (2016). Linkage mapping with paralogs exposes regions of residual tetrasomic inheritance in chum salmon (*Oncorhynchus keta*). *Molecular Ecology Resources*, *16*(1), 17–28. [https://doi.org/10.1111/1755-0998.12394](https://www.google.com/search?q=https://doi.org/10.1111/1755-0998.12394)

Zackheim, H. (2005). *A history of Montana Fish, Wildlife & Parks Fisheries Division 1901–2005*. Montana Fish, Wildlife & Parks. Helena, MT.

SUPPLEMENTARY TABLES

**Table S1.** Quantities of diagnostic SNP loci that distinguish species, subspecies, and orientations of interspecific and intraspecific inversions by the taxon for which they are informative. The chromosome 5 inversion complex had both interspecific and intraspecific diagnostic sets that were independent from one another; rainbow trout (RBT) subspecies loci are a single set of 94 markers that distinguish intraspecific inversion orientation. A similar notion applies to the RBT chromosome 20 (Omy20) intraspecific diagnostic loci. Taxa are coded as follows: RBT = rainbow trout; IRT = interior redband trout; CRT= coastal rainbow trout; WCT = westslope cutthroat trout; YCT = Yellowstone cutthroat trout.

| **Diagnostic Category** | **RBT** | **IRT** | **CRT** | **WCT** | **YCT** |
| --- | --- | --- | --- | --- | --- |
| Species | 12,413 | - | - | 5,650 | 7,407 |
| Subspecies | - | 118 | 51 | - | - |
| Chr05_Inv1 | 170 | 102 | 102 | 75 | 81 |
| Chr05_Inv2 | 131 | 94 | 94 | 78 | 61 |
| Chr17 | 35 | - | - | 16 | 19 |
| Chr20 | 168 | - | - | 45 | 47 |
| Omy20 | - | 22 | 22 | - | - |
| Chr22 | 134 | - | - | 53 | 43 |
| Chr29 | 60 | - | - | 17 | 25 |

Table S5. Comparison of pairwise *F*_ST_ between taxa across three distinct genomic partitions: the chromosome 5 inversion complex (Chr05 complex), the colinear non-inverted background of chromosome 5 (Chr05 background), and the rest of the genome (Genome-wide, excluding chromosome 5). *F*_ST_ was calculated in 500-kb non-overlapping windows. Descriptive statistics for *F*_ST_ are presented as Mean ± Standard Deviation (Median). The significance of the difference between the Chr05 complex and both background regions was assessed using a one-tailed Wilcoxon rank sum test for each taxon pair. The magnitude of these differences is reported as an effect size (Cliff’s Delta), followed by the *p*-value. Dashes (—) indicate baseline background regions against which the Chr05 complex was statistically compared. Taxa are coded as follows: CRT_AA_ = coastal rainbow trout (homozygous ancestral Chr05 genotype); CRT_RR_ = coastal rainbow trout (homozygous rearranged Chr05 genotype); IRT = interior redband trout; WCT = westslope cutthroat trout; YCT = Yellowstone cutthroat trout.

| **Taxon Pair** | **Region** | ***F*_ST_: Mean ± SD (Median)** | **# Windows** | **vs Genome: Effect (*p*)** | **vs Chr05: Effect (*p*)** |
| --- | --- | --- | --- | --- | --- |
| CRT_AA_ vs CRT_RR_ | Genome-wide | 0.063 ± 0.082 (0.053) | 3352 | — | — |
|  | Chr05 background | 0.081 ± 0.089 (0.078) | 69 | — | — |
|  | Chr05 Complex | 0.509 ± 0.174 (0.496) | 104 | 0.99 (<0.001) | 0.991 (<0.001) |
| CRT_AA_ vs IRT | Genome-wide | 0.214 ± 0.149 (0.198) | 3418 | — | — |
|  | Chr05 background | 0.189 ± 0.141 (0.176) | 70 | — | — |
|  | Chr05 Complex | 0.537 ± 0.18 (0.524) | 104 | 0.851 (<0.001) | 0.896 (<0.001) |
| CRT_AA_ vs WCT | Genome-wide | 0.511 ± 0.204 (0.521) | 3514 | — | — |
|  | Chr05 background | 0.469 ± 0.168 (0.468) | 70 | — | — |
|  | Chr05 Complex | 0.669 ± 0.201 (0.685) | 106 | 0.496 (<0.001) | 0.648 (<0.001) |
| CRT_AA_ vs YCT | Genome-wide | 0.555 ± 0.194 (0.555) | 3500 | — | — |
|  | Chr05 background | 0.508 ± 0.174 (0.496) | 70 | — | — |
|  | Chr05 Complex | 0.712 ± 0.166 (0.717) | 105 | 0.524 (<0.001) | 0.656 (<0.001) |
| CRT_RR_ vs IRT | Genome-wide | 0.214 ± 0.151 (0.2) | 3413 | — | — |
|  | Chr05 background | 0.194 ± 0.111 (0.182) | 67 | — | — |
|  | Chr05 Complex | 0.265 ± 0.164 (0.236) | 95 | 0.176 (0.002) | 0.243 (0.004) |
| CRT_RR_ vs WCT | Genome-wide | 0.505 ± 0.202 (0.514) | 3494 | — | — |
|  | Chr05 background | 0.471 ± 0.166 (0.476) | 70 | — | — |
|  | Chr05 Complex | 0.643 ± 0.21 (0.65) | 106 | 0.442 (<0.001) | 0.565 (<0.001) |
| CRT_RR_ vs YCT | Genome-wide | 0.548 ± 0.195 (0.548) | 3482 | — | — |
|  | Chr05 background | 0.503 ± 0.193 (0.506) | 71 | — | — |
|  | Chr05 Complex | 0.702 ± 0.178 (0.706) | 105 | 0.485 (<0.001) | 0.589 (<0.001) |
| IRT vs WCT | Genome-wide | 0.6 ± 0.215 (0.621) | 3483 | — | — |
|  | Chr05 background | 0.561 ± 0.193 (0.55) | 70 | — | — |
|  | Chr05 Complex | 0.67 ± 0.188 (0.698) | 106 | 0.207 (<0.001) | 0.358 (<0.001) |
| IRT vs YCT | Genome-wide | 0.644 ± 0.205 (0.658) | 3487 | — | — |
|  | Chr05 background | 0.589 ± 0.222 (0.595) | 72 | — | — |
|  | Chr05 Complex | 0.713 ± 0.188 (0.727) | 105 | 0.233 (<0.001) | 0.399 (<0.001) |
| WCT vs YCT | Genome-wide | 0.577 ± 0.233 (0.586) | 3432 | — | — |
|  | Chr05 background | 0.537 ± 0.218 (0.543) | 71 | — | — |
|  | Chr05 Complex | 0.606 ± 0.234 (0.635) | 105 | 0.097 (0.044) | 0.235 (0.004) |

SUPPLEMENTARY FIGURES

Figure S1. A graphical depiction of syntenic chromosomes between a. Arlee strain rainbow trout (representative of some coastal rainbow trout; CRT) with the homozygous ancestral arrangement (RBT_AA_) of the chromosome 5 inversion complex (Chr05 complex), b. Arlee strain CRT with the homozygous rearranged form (RBT_RR_) of the Chr05 complex, c. interior redband trout (IRT) native to the Kootenai River Basin, d. westslope cutthroat trout (WCT), and e. Yellowstone cutthroat trout (YCT). Haploid chromosome counts are listed with each taxon and chromosome numbers are presented in black for RBT taxa, red for WCT, and green for YCT. Chromosomes harboring interspecific or intraspecific inversions are rotated 90 degrees relative to chromosomes without inversions to illustrate the approximate location of inversions and their synteny with other taxa. Inversions are denoted as a segment of a chromosome bounded by vertical dotted lines. Hatched patterns across the inversion region denote whether a taxon carries the rearranged/derived form of the inversion (R); a lack of hatched patterning indicates the presence of the ancestral form of the inversion (A). Crossed dotted lines connecting two taxa at an inversion denote that they are inverted relative to one another. This figure is adapted from Figure S1 in Flores et al. (2025) with chromosome numbers and the inferred karyotype of YCT being derived from their work. This figure was created using BioRender.

Figure S2. A visual representation of the SNPs used in this study and their positions in the Arlee strain Rainbow Trout genome (NCBI: GCF_013265735.2; Gao et al. 2021). Light gray bars onto which tick marks are plotted represent the full physical extent of each chromosome. Sets represented include a. all 189,984 SNPs and diagnostic SNP sets for b. rainbow trout (*Oncorhynchus mykiss*; RBT), c. westslope cutthroat trout (*O. lewisi*; WCT), d. Yellowstone cutthroat trout (*O. virginalis bouvieri*; YCT), e. interior redband trout (*O. m. gairdneri*; IRT), and f. coastal rainbow trout (*O. m.* ssp.; CRT). The set and number of markers are specified on each plot.

**Figure S3.** Manhattan plots of pairwise Weir and Cockerham’s *F*_ST_ calculated in non-overlapping 500-kb windows using VCFtools with inversion-aware reference groups, subset to equal sample sizes (*N* = 31). Comparisons are shown for coastal rainbow trout (CRT) with homozygous ancestral and derived Chr05 complex genotypes (CRTAA and CRTRR), interior redband trout (IRT), westslope cutthroat trout (WCT), and Yellowstone cutthroat trout (YCT).

**Figure S4.** Alignment of the global consensus IRT genetic map to the **a.** Arlee RBT (NCBI: GCF_013265735.2; Gao et al. 2021), **b.** Swanson RBT (NCBI: GCA_025558465.1; Ali et al. 2025), and **c.** Connor Lake WCT (NCBI: GCF_045791955.1; Flores et al. 2025) reference genomes. For each IRT linkage group (LG01–LG29), genetic position in the global consensus map (cM; *y*-axis) is plotted against physical position on the corresponding reference chromosome (Mb; *x*-axis). Each point represents a mapped marker aligned to the reference genome; blue and red points indicate alignments to the positive (+) and negative (-) strands, respectively. Differences in marker order, orientation, and correspondence among linkage groups and reference chromosomes reveal karyotypic differences and candidate structural rearrangements among genomes. Chromosome labels follow the nomenclature of the respective reference genome. Complete chromosome-by-chromosome plots for panels **a**–**c** are provided as separate supplementary PDF files accompanying this document (**Figure S4a–c**).

Figure S5. Comparisons of ancestry estimates with bar graphs for a. pairwise Pearson correlation coefficient (*r*) values and b. pairwise root mean square error (RSME) values comparing each global ancestry method result for each ancestry category examined in this study: Diagnostics = normalized proportions derived from species- and subspecies-diagnostic locus sets; ADMIXTURE = supervised ADMIXTURE v1.3.0 (Alexander et al. 2009); ELAI = global ancestry proportions derived from the program Efficient Local Ancestry Inference (ELAI) v1.01 (Guan 2014). Taxa for which proportions were generated are coded as follows: coastal rainbow trout = CRT; IRT = interior redband trout; westslope cutthroat trout = WCT; Yellowstone cutthroat trout = YCT.

**Figure S6.** Sensitivity of hybrid classification to linkage disequilibrium pruning. Hybrid index (proportion of RBT ancestry using RBT species-diagnostic loci) plotted against interspecific heterozygosity for RBT×WCT hybrids using an LD-pruned diagnostic SNP panel. LD pruning retained 103 of 11,071 overlapping RBT-diagnostic SNPs. Hybrid index and interspecific heterozygosity remained highly correlated with estimates from the unpruned dataset (*r* = 0.9998 and *r* = 0.9895, respectively), and all eight identified F1 individuals retained intermediate hybrid indices and high interspecific heterozygosity. Classification criteria are identical to those used for Figure 3.

**Figure S7.** Chromosome painting plots showing the population-level consensus of ancestry blocks—excluding homozygous inland redband trout (IRT)—estimated by the program Efficient Local Ancestry Inference (ELAI) (Guan 2014) across all individuals in two collections from lower Bear Creek in the Libby Creek Drainage: **a.** Bear_20 and **b.** Bear_21. Ancestry blocks are mapped to the Arlee strain rainbow trout reference genome (NCBI: GCF_013265735.2; Gao et al. 2021) and the total length of each chromosome is represented by an underlying black dotted line. Homozygous coastal rainbow trout (CRT) and westslope cutthroat trout (WCT) ancestry blocks are found in some portions of the genome, but hybrid ancestry primarily occurred as heterozygous blocks. Interspecific inversions are depicted as hollow black boxes and include the Chromosome 5 (Omy05) complex which displays intraspecific polymorphism between CRT and IRT. Despite low global CRT ancestry proportions (3.80% in Bear_20 and 4.66% in Bear_21), much of the genome is affected by CRT hybridization across both collections.

**Figure S8.** Scatterplots showing the relationship between inversion-specific heterozygosity (x-axis) and chromosome-wide heterozygosity excluding the inversion (y-axis) at rainbow trout (RBT) diagnostic loci. Pearson correlation coefficients (*r*) are shown. Points are colored by inferred hybrid class with hybrids beyond the F1 (RBT×WCT) hybrid class noted as a backcross with majority RBT, westslope cutthroat trout (WCT), or Yellowstone cutthroat trout (YCT) ancestry. Relative to RBT, WCT have a fusion of RBT chromosomes 20 (Omy20) and 28 (Omy28) which forms WCT chromosome Ocl20 (20/28 fusion). This fusion also exists in YCT (Ostberg et al. 2013; Flores et al. 2025). These plots show evidence of recombination suppression across Omy28 when the Chr20 interspecific inversion is present in hybrid individuals that are Robertsonian heterozygotes. The Chr20 inversion was already shown to suppress recombination across Omy20 in Figure 3 (Pearson’s *r* = 0.96), indicating that this inversion and Robertsonian heterozygosity may both contribute to strong recombination suppression across both RBT chromosomes in interspecific hybrids. In contrast, Chr20 inversion heterozygosity was not associated with recombination suppression on chromosome 10 (Chr10), which has no associated fusion. Evidence of free recombination on Chr10 was observed regardless of Chr20 inversion presence.

**Figure S9.** Scatterplots showing the relationship between heterozygosity across rainbow trout (RBT) chromosome arm 1 (x-axis) and RBT chromosome arm 2 (y-axis) at RBT-diagnostic loci for those RBT chromosomes that correspond to fissions in westslope cutthroat trout (WCT) as seen in Figure S1. Pearson correlation coefficients (*r*) for each structural variant are shown. Points are colored by inferred hybrid class with hybrids beyond the F1 (RBT×WCT) hybrid class noted as a backcross with majority RBT, westslope cutthroat trout (WCT), or Yellowstone cutthroat trout (YCT) ancestry. Recombinants were observed at a lower frequency for these RBT chromosomes than for chromosome 1 (Chr01), which is not associated with rearrangements in cutthroat trout relative to RBT. Heterozygosity across arm 1 was strongly associated with heterozygosity across arm 2 for all WCT fissions, indicating that Robertsonian heterozygotes in hybrids are likely resulting in recombination suppression but it is weaker than suppression from interspecific inversions.

**Figure S10.** Chromosome painting plots showing the population-level consensus of ancestry blocks—excluding homozygous interior redband trout (IRT)—estimated by the program ELAI (Guan 2014) across all individuals in two IRT populations believed to be naturally introgressed with westslope cutthroat trout (WCT) and isolated from nonnative taxa: **a.** upper East Fork Yaak River (EF_Yaak_20, EF_Yaak_21, EF_Yaak_22, and EF_Yaak_24) and **b.** upper Bear Creek (Bear_17, Up_Bear_22, and Bear_24). Blocks are mapped to the Arlee strain rainbow trout reference genome (NCBI: GCF_013265735.2; Gao et al. 2021). Interspecific inversions are depicted as hollow black boxes. The EF Yaak consensus displays admixture that affects the majority of the genome and occurs in large, contiguous blocks driven by recent, ongoing hybridization. In contrast, the Upper Bear consensus exhibits a highly fragmented pattern typical of historical introgression, where trace IRT×WCT ancestry (IRT/WCT heterozygous blocks) is scattered across colinear regions but is completely absent within interspecific inversions.

**Figure S11.** Relationship between the number of interspecific heterokaryotypic inversions and genome-wide admixture proportions in Kootenai Basin *Oncorhynchus* hybrids. Minor parent ancestry (x-axis)—calculated as the minimum of the rainbow trout (RBT) or westslope cutthroat trout (WCT) ELAI ancestry proportions—serves as a proxy for the extent of backcrossing. The y-axis displays the per-individual count of heterokaryotypic inversions (0–6), explicitly separating the Chr05 double inversion complex into two independent variants alongside Chr17, Chr20, Chr22, and Chr29. **a.** Jittered scatterplot of the continuous relationship between minor parent ancestry and inversion count, with a LOESS regression line (gray shading indicates 95% confidence interval). **b.** Boxplots displaying the distribution of heterokaryotypic inversions binned by admixture categories representing extent of backcrossing, overlaid with raw data points. Both panels illustrate the loss of structural heterozygosity during repeated backcrossing.
